# Meta-Analysis on the Chinese Herbal Formula *Xiaoer-Feike Granules* as a Complementary Therapy for Children With Acute Lower Respiratory Infections

**DOI:** 10.3389/fphar.2020.496348

**Published:** 2020-10-22

**Authors:** Qiang You, Lan Li, Dan Li, Dan Yang, Lin Chen, Hong-ping Chen, You-ping Liu

**Affiliations:** ^1^School of Pharmacy, Chengdu University of Traditional Chinese Medicine, Chengdu, China; ^2^The Affiliated Hospital of Southwest Medical University, Luzhou, China; ^3^School of Nursing, Southwest Medical University, Luzhou, China

**Keywords:** Chinese herbal formula, *Xiaoer-Feike granules*, acute lower respiratory infection, meta-analysis, randomized controlled trials

## Abstract

**Background:**

Over the past five years the Chinese herbal formula (CHF) medicine, *Xiaoer-Feike granules* (XFG), has become a widely used adjuvant therapy for acute lower respiratory infections (ALRI). Considering the rapid popularization and application of XFG, and the lack of systematic evidence evaluating its effectiveness and safety in treating ALRI, it is necessary to conduct a meta-analysis to determine its benefits for patients.

**Methods:**

This study systematically identified randomized controlled trials (RCTs) of XFG treatments for ALRI through July 2019 using four English-databases (PubMed, Cochrane Library, Ovid, and Web of Science) and four Chinese-databases (Sino-med database, China National Knowledge Infrastructure (CNKI), VIP database, and the WANFANG database). We then performed a quality assessment and data analysis with Review Manager 5.3.5 and Stata 15.1.

**Results:**

Twenty-one RCTs involving 3425 patients were randomly divided into an XFG group and a conventional medicine (CM) group. The results showed that the clinical efficacy rate (CER) of the XFG group was significantly higher than that of the CM group (*RR*=1.17, 95% *CI* =1.13-1.22, *P*< 0.00001). In comparison with the CM group, the XFG group had strikingly shortened: resolution time of cough (RTC) (*MD* = -1.92; 95% *CI* =-2.33, -1.51, *P*<0.00001); resolution time of rale (RTR) (*MD* = -1.68; 95% *CI* =-2.27, -1.10, *P*<0.00001); resolution time of fever (RTF) (*MD* = -1.46; 95% *CI* =-1.92, -1.00, *P*<0.00001); resolution time of inflammatory lesions (RTIL) (*MD* = -2.43, 95% *CI* =-2.94, -1.93, *P*< 0.00001); and hospital stays (HS) (*MD* = -2.26, 95% *CI* =-3.03, -1.49, *P*< 0.00001). At the cellular and molecular level, the CD4, CD8, CD4/CD8, IL-6, TNF-α, and CRP levels were significantly improved when CM was complemented with XFG. In addition, no significant difference was observed between the XFG and CM groups in terms of the adverse events (AE) (*RR* =0.97, 95% *CI=* 0.61-1.54, *P=* 0.89).

**Conclusions:**

The findings of this meta-analysis support the use of XFG in the treatment of ALRI. However, these results should be treated with caution due to the significant heterogeneity and publication bias of existing data. Further well-designed and high-quality RCTs are needed to interrogate the efficacy and safety of XFG.

## Introduction

Acute lower respiratory infections (ALRI), primarily including pneumonia and bronchiolitis with typical clinical symptoms such as fever, cough, asthma, rale, and so forth, are among the most severe infectious diseases (Emam et al., 2019). ALRI leads to nearly one million deaths in children under five years of age every year, a much higher rate than all other childhood illnesses (UNICEF/WHO. UNICEF, 2016). Among ALRIs, pneumonia is the number one killer of children. In particular, ALRI primarily affects patients in developing countries due to limited health care and poorer control of infection risk factors. In some parts of the world, one child dies of pneumonia every 35 seconds, and the combined number in developing countries accounts for more than 99% of childhood pneumonia deaths (UNICEF/WHO. UNICEF, 2006). Despite a significant decline in child deaths from ALRI between 2000 and 2015, 19% of all childhood deaths were still attributable to ALRI (Bryce et al., 2005; Rudan et al., 2008). Children with ALRI not only endure a lower quality of life, these conditions can also cause significant medical and financial burdens on families and society (Jiang et al., 2016).

The pathogenic microorganisms of ALRI include bacteria *(Streptococcus pneumoniae, Staphylococcus aureus, Escherichia coli, Klebsiella Pseudomonas aeruginosa*, etc.), fungi (*Candida albicans* and aspergilloma), viruses (RSV, IFV, IFB, and CMV), mycoplasma, *Chlamydia trachomatis, Rickettsia*, and protozoal pneumonia. Bacterial and viral infections predominate ALRI. Currently, conventional medicines (CM) consist of defervescence, antitussive, expectorant (ambroxol and clenbuterol), aerosol, and anti-infection agents along with a variety of antibiotics, such as ceftriaxone sodium, ceftizoxime sodium, levofloxacin, and azithromycin, which have shown results in reducing ALRI mortality (He and Yang, 2018). However, because patients are often desperate for a speedy recovery, they are often irrationally prescribed antibiotics. This overuse of antibiotics worldwide has gradually enhanced the drug resistance of ALRI pathogens, which has significantly increased ALRI infection rates (Zhang L. et al., 2018). In the long run, the benefits from CM as led by antibiotics are likely to be challenged. Thus, research on novel therapies that can substantially enhance the effect of CM and reduce antibiotic use is of great social and clinical importance.

In recent years increasing numbers of parents in East Asia have sought complementary and alternative medicine for their children. In China, Chinese herbal formulas (CHFs), based on the basic theories of traditional Chinese medicine (TCM), are widely accepted and prescribed for children with ALRI. For example, XFG was first recorded in the 2015 edition of the Chinese pharmacopoeia and is one of the most effective CHFs used for ALRI. XFG consists of 22 ingredients: Ginseng radix et rhizoma, Poria, *Atractylodis macrocephalae* rhizoma, Citri reticulatae Pericarpium, Rhei radix et rhizome, Lycii cortex, Glehniae radix, Glycyrrhizae radix et rhizome, *Artemisiae annuae* herba, Ophiopogonis radix, Cinnamomic ramulus, Zingiberis rhizoma, Aconiti radix, Trichosanthis Fructus, Farfarae flos, Asteris radix et rhizome, Mori cortex, Astragali radix, Lycii fructu, Arisaema cum Bile, Galli gigerii endothelium corneum, and Trionycis carapax. XFG exerts its therapeutic effects mostly by inhibiting pathogenic microorganisms, enhancing immune function, reducing inflammation, relieving bronchospasm, and improving digestion and appetite (Chu, 2018; Hu et al., 2018; Wei and Li, 2018). Increasing numbers of clinical studies have indicated that XFG provides more benefits to preschool children with ALRI than CM alone (Yan, 2017). There is abundant evidence demonstrating that XFG can significantly shorten the duration of cough, asthma, fever, rale, hospital stays (HS), and antibiotic use and promote recovery from ALRI (Zhao and Chen, 2017; Liao and Lou, 2018). However, considering the rapid popularization and application of XFG, and the lack of systemic evidence to support the effectiveness and safety of XFG in treating ALRL, it is necessary to conduct a meta-analysis and rigorously assess the efficacy and safety of XFG to provide more objective and reliable evidence for the use of XFG in clinical settings.

## Methods

### Search Strategy

The databases we searched in this review included PubMed, WANFANG, Ovid, Web of Science, Cochrane Library, China National Knowledge Infrastructure (CNKI), VIP database, Sino-med database through July 2019. Considering that XFG was primarily used in China, we searched the above Chinese-language electronic databases to obtain as many clinical trials as possible. The search was restricted to trials published in Chinese and English. For the English-language databases, we used the following search strategies: Subject terms= (“Feike” or “pediatric lung cough” or “pediatric pulmonary cough”). For the Chinese databases, we used Subject terms= (“Xiaoer-Feike”). We also manually searched for articles that met our inclusion criteria from other sources that were not included in the above databases. Eligible studies were screened out by two reviewers independently. When a discrepancy occurred between the two investigators, it was resolved by discussion.

### Article Inclusion and Data Extraction

This systematic review was conducted according to the Preferred Reporting Items for Systematic Review and Meta-Analyses Statement (PRISMA). We selected eligible studies based on the following inclusion criteria: (1) the experimental group was treated with the XFG included in the Chinese Pharmacopoeia (edition 2015) regardless of the pharmaceutical company, (2) the control group was treated with conventional medicines alone, and (3) the clinical efficacy rate (CER), resolution time of cough (RTC), resolution time of rale (RTR), resolution time of fever (RTF) and HS were used as the primary outcomes by referring to guidelines or consensus views or the evaluation criteria. (4) In addition to the CER, at least one other outcome was employed. The exclusion criteria were: (1) non-RCTs or only one outcome; (2) the XFG group received the combination treatment of XFG and acupuncture therapy or other TCM; (3) studies that did not have control groups, or studies in which control subjects received TCM treatment including herbal medicine, acupuncture, or acupoint injection therapy; (4) the XFG that was not recorded in the Chinese Pharmacopoeia; and (5) systematic review, important data reports, and case reports were addressed. Two reviewers independently checked for relevant literature, including the first author’s last name, year of publication, sample size, gender distribution, age, disease duration, intervention, duration of treatment, dose, outcomes and adverse events, and clinical diagnosis.

### Quality Assessment

Two reviewers independently evaluated the methodological qualities of the trials according to the Cochrane manual. We identified a list of seven categories for risk of bias: selection bias, performance bias, detection bias, attrition bias, reporting bias, and other bias. Each item was classified into low bias risk, high bias risk, and unclear bias risk. Disagreements between the reviewers were settled through discussion.

### Data Synthesis and Analysis

In this review, statistical analyses were conducted by a reviewer manager (version 5.3.5), and we used OR with 95% *CI* for the analyses of dichotomous data, whereas the continuous data were presented as MD or sMD with 95% *CI*. The data were merged according to the Mantel-Haenszel (fixed-effects) model and the DerSimonian and Laird (random-effects) model. The heterogeneity between studies was determined by the chi-square test. With the *I*^2^ statistic, an *I*^2^< 25% indicating that heterogeneity may not be important, values between 25% and 50% represent moderate inconsistency, and *I^2^* > 50% suggest severe heterogeneity. We defined *P*≥ 0.1 and *I*^2^< 50 as indications that the results are consistent and that the fixed-effects model would be used, while *I*^2^> 50% was an indicator of significant heterogeneity among trials. Then, a random-effects model was used to pool the results to minimize the influence of potential clinical heterogeneity. We evaluated the robustness of publication bias and sensitivity analyses to evaluate the robustness of the merged results with Stata 15.1. All the statistical tests were two tailed, and the differences were statistically significant at *P*< 0.05.

## Results

### Search Results and Study Characteristics

**Figure 1:**
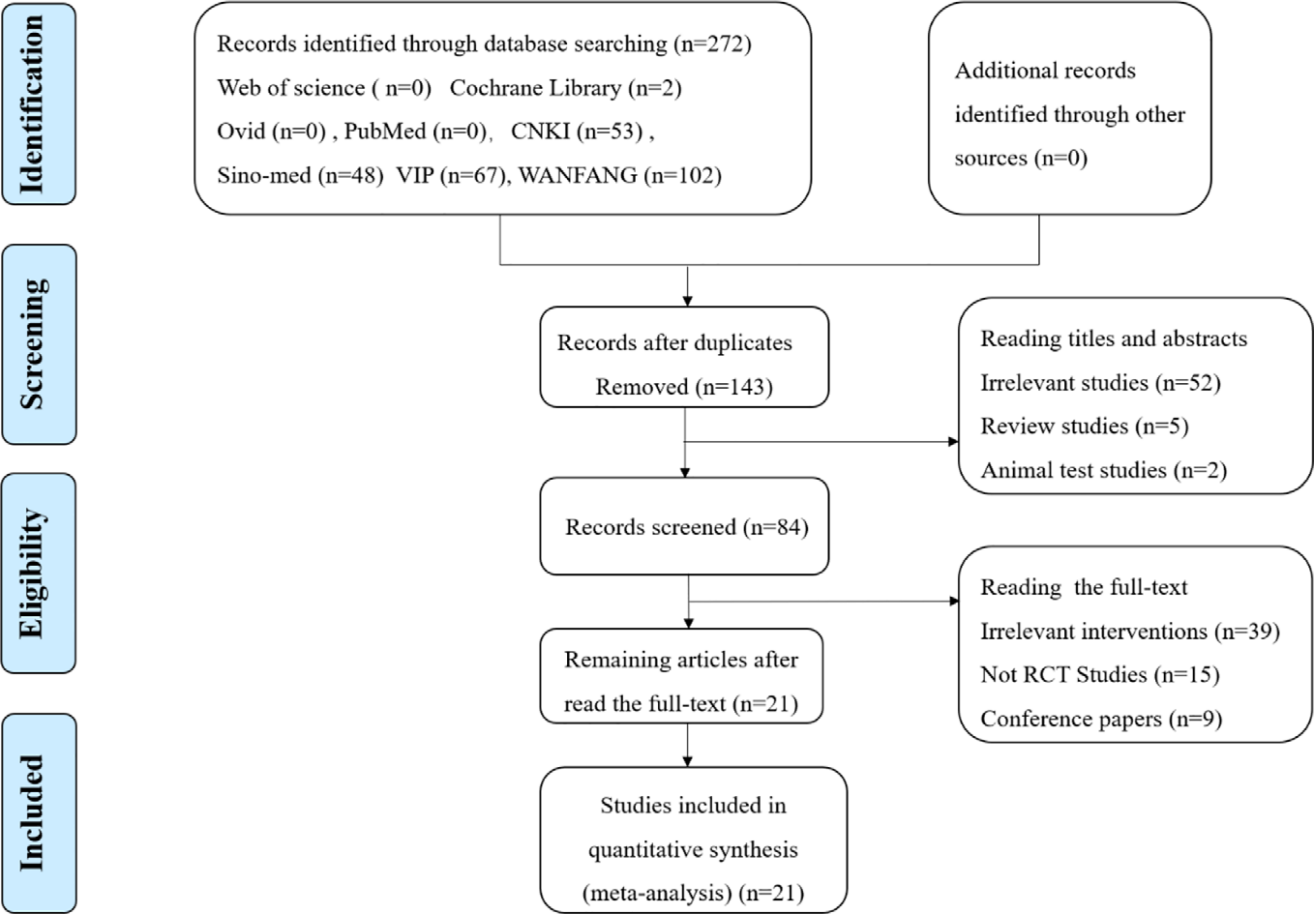
Flow chart of study selection.

A total of 272 papers were obtained from a database search of the following sources: Web of Science (n=0), Cochrane Library (n=2), Ovid (n=0), PubMed (n=0), CNKI (n=53), Sino-med (n=48), VIP (n=67), WANFANG (n=102), from which 129 duplicated publications were removed. Fifty-nine citations of irrelevant topics were excluded after reading the titles and abstracts (irrelevant studies (n=52), review studies (n=5), and animal test studies (n=2)), and 63 studies were ruled out following a screening of the full text (irrelevant interventions (n=39), not RCT studies (n=15), and conference papers (n=9). Finally, 21 RCTs published between 2014 and 2018 involving 3425 patients with ALRI were eligible according to the inclusion criteria. Two reviewers extracted data from the literature, including information on general trial characteristics (first author’s last name, publication date); baseline patient and disease data (number of patients in each group, age and disease course); interventions (XFG, conventional medicine, treatment duration, and dose), outcome definitions, detailed adverse reactions, and clinical diagnosis. The sample size was 60 to 600 with significant age differences. The treatment duration ranged from 5 to 14 days. The XFG was provided by two Chinese pharmaceutical companies, Tian-sheng Pharmaceutical Group co., LTD, and Chang-chun People Pharmaceutical g=Group co., LTD. Twelve trials reported a specific number of adverse events. Of the 21 trials, 14 included patients diagnosed with acute bronchitis, and the other 7 studies selected pneumonia patients. Generally, the basic characteristics of the two groups were consistent, and no significant difference was found before the intervention. The procedure of the literature search is shown in **Figure 1**, and the general characteristics of the selected studies are listed in **Table 1**.

**Table 1:** The baseline Characteristics of the 21 studies.

Study	Sample size			Age/disease course(day)			Intervention		Treatment duration	Outcome	Adverse events	Clinical diagnosis
	E(M/F)		C(M/F)	E		C	E	C				
Chu, 2018	60 (28/32)	60 (27/33)		8.1 ± 1.7/ 3.5 ± 1.2	6.7 ± 2.1/ 3.3 ± 1.0		Xiaoer-Feike granules 5-6g, bid, PO Conventional medicines	Conventional medicines	14d	CER, RTC, RTR	E: nausea (1), diarrhea (2) C: nausea (2), diarrhea (2) emesis (1)	acute bronchitis
Du, 2016	43 (20/23)	43 (21/22)		5.94 ± 2.08/ 1.19 ± 0.42	5.98 ± 2.37/ 1.18 ± 0.43		Xiaoer-Feike granules Ages: 0-1(2g); 1-4(3g); 5-8(6g), tid, PO Conventional medicines	Conventional medicines	7d	CER, TLSL	no serious adverse reactions	acute bronchitis
Kong, 2016	191 (106/85)	191 (108/83)		5.2 ± 1.3/ ─	5.1 ± 1.2/ ─		Xiaoer-Feike granules Ages: 1-3(6g); 4-7(9g); 8-11(12g), tid, PO Conventional medicines	Conventional medicines	5d	CER, RTC, RTA	unclear	acute bronchitis
Liu, 2015	63 (32/31)	62 (32/30)		1.6 ± 0.72/ ─	1.6 ± 0.70/ ─		Xiaoer-Feike granules Ages: 0-1(2g); 1-4(3g); 5-8(6g), tid, PO Conventional medicines	Conventional medicines	7d	CER, RTC, RTR, RTF, RTIL, RTAU, HS	E: mild diarrhea (2)	acute bronchitis
Ma and Lu, 2017	85 (41/44)	85 (45/40)		5.5 ± 2.4/ 35.7 ± 11.5	5.2 ± 2.9/ 36.5 ± 10.3		Xiaoer-Feike granules Ages: 0-3(3g); 4-6(3g); 6-12(9g), tid, PO Conventional medicines	Conventional medicines	14d	CER, RTC, CGRP, Leukotrienes, TNF-α, IL-8	E: dizziness and headache (2), gastrointestinal discomfort (2) C: dizziness and headache (3), gastrointestinal discomfort (2)	acute bronchitis
Qian and Li, 2018	98 (51/47)	95 (49/46)		1.72 ± 1.2/ <1	1.61 ± 1.1/ <1		Xiaoer-Feike granules (2g/Kg, tid, PO) Conventional medicines	Conventional medicines	5d	CER, RTC, RTR,	unclear	acute bronchitis
Shang, 2015	44 (-/-)	42 (-/-)		6.11 ± 1.04/ 2-12	6.13 ± 1.03 2-12		Xiaoer-Feike granules Ages: 0-1(2g); 1-4(3g); 5-8(6g), tid, PO Conventional medicines	Conventional medicines	7d	CER, RTC, RTR, RTF, RTA	unclear	acute bronchitis
Wei and Li, 2018	46 (23/23)	43 (22/21)		3.7 ± 2.1/ ─	4.7 ± 2.3/ ─		Xiaoer-Feike granules Ages: 0-1(2g); 1-4(3g); 5-8(6g), tid, PO Conventional medicines	Conventional medicines	7d	CER, RTC	no adverse reactions	acute bronchitis
Wang and Guo, 2014	200 (93/107)	80 (41/39)		5.03 ± 2.45/ 6.87 ± 3.79	4.97 ± 2.67/ 7.11 ± 4.11		Xiaoer-Feike granules Ages: 0-1(2g); 1-4(3g); 5-8(6g), tid, PO Conventional medicines	Conventional medicines	8d	CER, RTC, RTR, RTA	unclear	acute bronchitis
Xiao and Li, 2016	35 (18/17)	35 (-/-)		0.7-10/ 4.52 ± 2.50	0.7-10/ 4.71 ± 2.48		Xiaoer-Feike granules Ages: 0-1(2g); 1-4(3g); 5-7(6g), tid, PO Conventional medicines	Conventional medicines	7d	CER, RTC, RTR, RTF	unclear	acute bronchitis
Yan, 2015	50 (-/-)	50 (-/-)		1-11 1-4	1-11/ 1-4		Xiaoer-Feike granules Ages: 1-4(3g); 5-11(6g); tid, PO Conventional medicines	Conventional medicines	5-7d	CER, RTF, RTC, RTR	no adverse reactions	acute bronchitis
Yan, 2017	45 (24/21)	45 (23/22)		0.4-11/ 4.49 ± 1.95	0.5-11/ 4.51 ± 1.86		Xiaoer-Feike granules Ages: 0-1(2g); 1-4(3g); 5-8(6g), tid, PO Conventional medicines	Conventional medicines	7d	CER, RTC, RTF, RTR, HS	E: mild diarrhea (2)	acute bronchitis
Zhang J. Q. et al., 2018	40 (16/24)	40 (24/16)		6.13 ± 4.01/ 1.30 ± 0.41	5.16 ± 4.05/ 1.19 ± 0.36		Xiaoer-Feike granules Ages: 0-1(2g); 1-4(3g); 5-8(6g), tid, PO Conventional medicines	Conventional medicines	7d	CER, TLSL	no serious adverse reactions	acute bronchitis
Zhang and Guo, 2018	47 (26/21)	46 (24/22)		5.27 ± 2.94/ ─	5.41 ± 2.92/ ─		Xiaoer-Feike granules Ages: 3-6(3g); 6-9(6g); 9-12(9g), tid, PO Conventional medicines	Conventional medicines	14d	CER, CRP, IL-4, TNF-α, INF-γ, LCQ	E: gastrointestinal discomfort (3), electrolyte disturbance (1), headache (1) C: gastrointestinal discomfort (3), electrolyte disturbance (2), headache (1), dizziness (1)	acute bronchitis
He and Yang, 2018	80 (45/35)	70 (39/31)		2.61 ± 0.72/ 4.92 ± 1.51	2.59 ± 0.70/ 5.06 ± 1.57		Xiaoer-Feike granules Ages: 0-1(2g); 1-4(3g); 5-8(6g), tid, PO Conventional medicines	Conventional medicines	5-7d	CER, RTC, RTF, RTR, HS	E: diarrhea (6) C: diarrhea (5)	pneumonia
Hu et al., 2018	54 (32/22)	54 (28/26)		7.81 ± 1.24/ 1.78 ± 0.25	7.69 ± 1.18/ 1.74 ± 0.23		Xiaoer-Feike granules Ages: 3-4(3g); 5-12(6g), tid, PO Conventional medicines	Conventional medicines	7d	CER, RTC, RTR, RTF, MEL, IL-6, IGF-II, CRP	no adverse reactions	pneumonia
Li, 2015	300 (168/132)	300 (174/126)		2.25 ± 0.24/ 10.19 ± 1.17	2.17 ± 0.22/ 10.52 ± 1.22		Xiaoer-Feike granules (3g, tid, PO) Conventional medicines	Conventional medicines	14d	CER, RTF, RTC, CRP, WBC	unclear	pneumonia
Liao and Lou, 2018	53 (29/24)	53 (27/26)		4.41 ± 1.43/ ─	4.47 ± 1.18/ ─		Xiaoer-Feike granules Ages: 0-1(2g); 1-4(3g); 5-8(6g), tid, PO Conventional medicines	Conventional medicines	14d	CER, RTF, RTC, RTIL, APC, IL-1R1	E: gastrointestinal discomfort (5) C: gastrointestinal discomfort (6)	pneumonia
Xu et al., 2015	31 (20/11)	30 (19/20)		4.1 ± 1.5/ ─	4.3 ± 1.2/ ─		Xiaoer-Feike granules Ages: 0-1(2g); 1-4(3g); 5-8(6g), tid, PO Conventional medicines	Conventional medicines	14d	CER, RTC, RTF, RTIL	mild gastrointestinal discomfort	pneumonia
Ye and Zhen, 2016	32 (17/15)	30 (16/14)		4.6 ± 1.4/ ─	4.5 ± 1.2/ ─		Xiaoer-Feike granules Ages: 0-1(2g); 1-4(3g); 5-8(6g), tid, PO Conventional medicines	Conventional medicines	14d	CER, RTC, RTR	E: nausea (1), diarrhea (1) C: diarrhea (2)	pneumonia
Zhao and Chen, 2017	200 (112/88)	200 (109/91)		4.4 ± 2.2/ 6.2 ± 3.9	4.8 ± 2.8/ 7.0 ± 4.2		Xiaoer-Feike granules Ages: 1-4(3g); 5-13(6g), tid, PO Conventional medicines	Conventional medicines	5d	CER, RTC, RTF, RTR, TNF-α, IL-6, IL-10, HMGB1	E: nausea (2), rash (1) C: rash (2)	pneumonia

E, experiment group; T, control group; TLSL, T-lymphocyte subpopulation level; RTIL, resolution time of inflammatory lesions; RTA, resolution time of asthma; RTAU, resolution time of antibiotic use; CGRP, calcitonin gene related peptides; APC, antigen-presenting cells; CRP, C-reactive protein; LCQ, Leicester cough questionnaire; MEL, myocardial enzymes level; HMGB1, high mobility group box-1 protein.

### Methodological Quality Assessment

**Figure 2:**
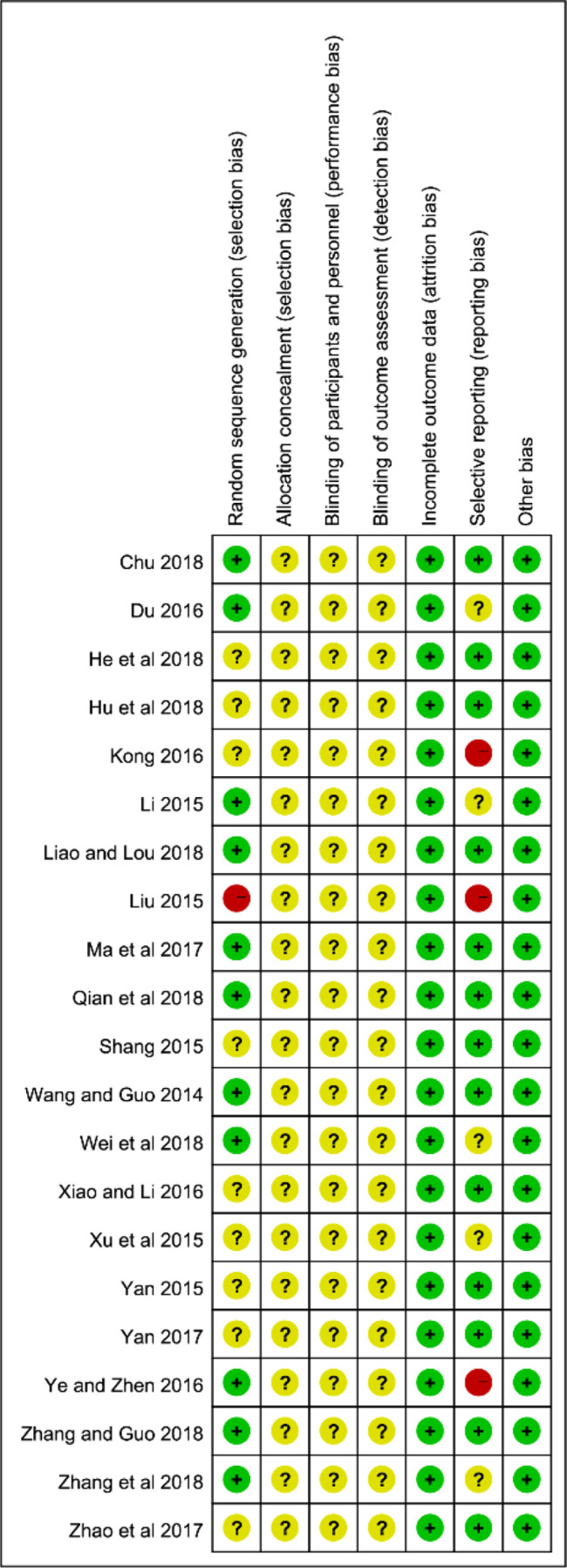
Assessment of risk of bias in the 21 trials.

The specific randomized methods were detailed in 11 studies by random number tables in the assessments of selection bias, and we considered them as low-risk (Wang and Guo, 2014; Li, 2015; Du, 2016; Ye and Zhen, 2016; Ma and Lu, 2017; Chu, 2018; Liao and Lou, 2018; Qian and Li, 2018; Wei and Li, 2018; Zhang and Guo, 2018; Zhang J. Q. et al., 2018). One study (Liu, 2015) was high-risk due to its use of the registration order to generate a random sequence. The remaining 9 articles did not offer any specific information regarding the generation of random sequences. Almost all the studies failed to give the specific allocation concealment, performance bias, and detection bias. The reporting bias was at high risk in 3 documents (Liu, 2015; Kong, 2016; Ye and Zhen, 2016) because they failed to report the pre-listed outcomes. On the whole, the 21 studies fell in the middle because of the high proportion of the unclear risk of biases in the majority of studies. The specific results of the bias assessment are summarized in **Figure 2**.

#### Primary Outcomes

##### CER

**Figure 3:**
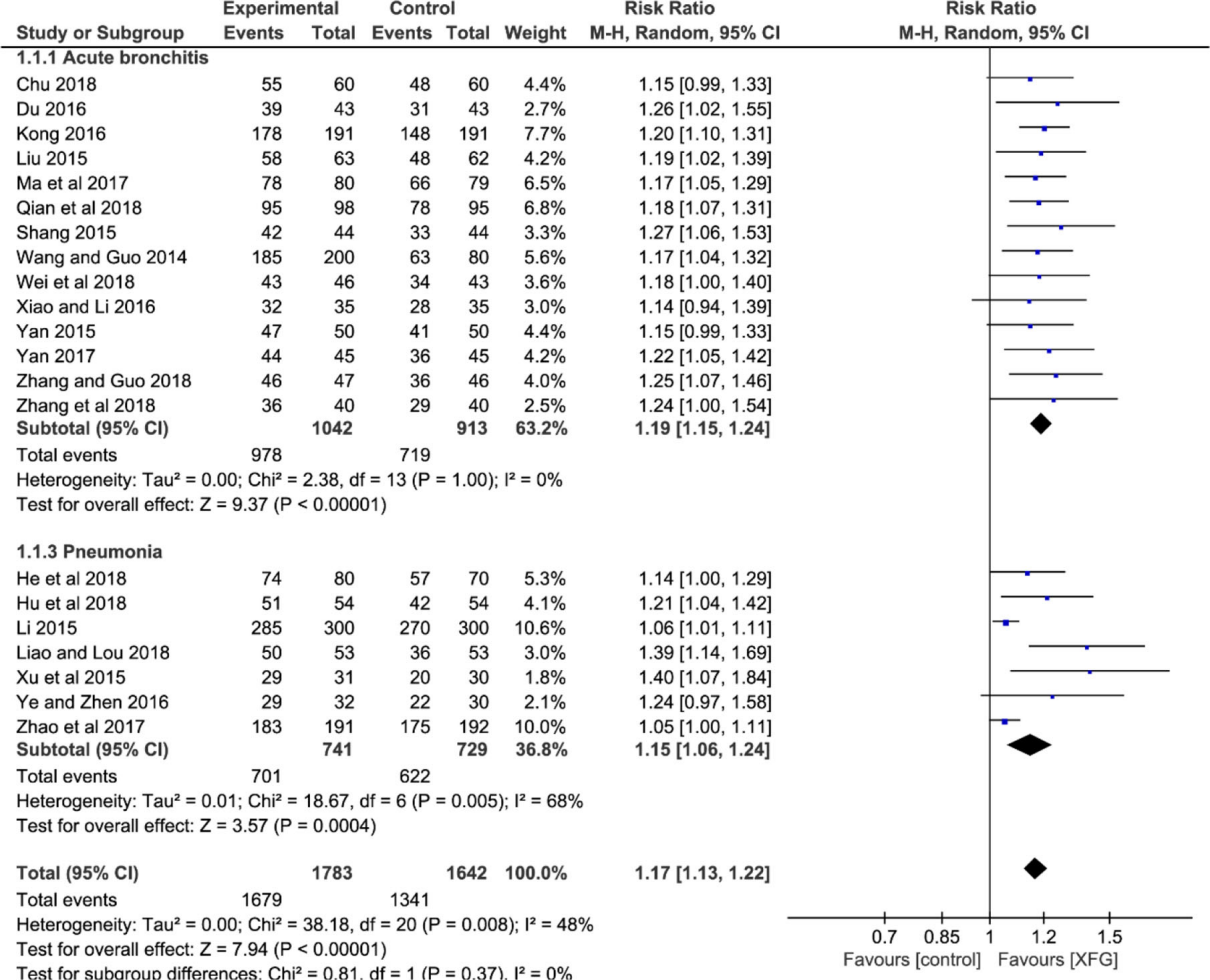
Forest plot and subgroup analysis of the CER.

All the studies reported CER involving 1783 patients in the experimental groups who were treated with a combination of XFG and CM, and 1642 patients in the control groups were treated with CM alone. The heterogeneity test suggested that the fixed-effects model was more suitable (*P*=0.008, *I^2^* = 48%). The result showed a statistically significant difference between the two groups, which indicated that adding XFG provided more benefits to ALRI patients. (*RR*=1.17, 95% *CI* =1.13-1.22, and *P*< 0.00001), and no significant difference was found between acute bronchitis and pneumonia groups (*P*= 0.37, *I^2^* = 0%), as shown in **Figure 3**.

##### RTC

**Figure 4:**
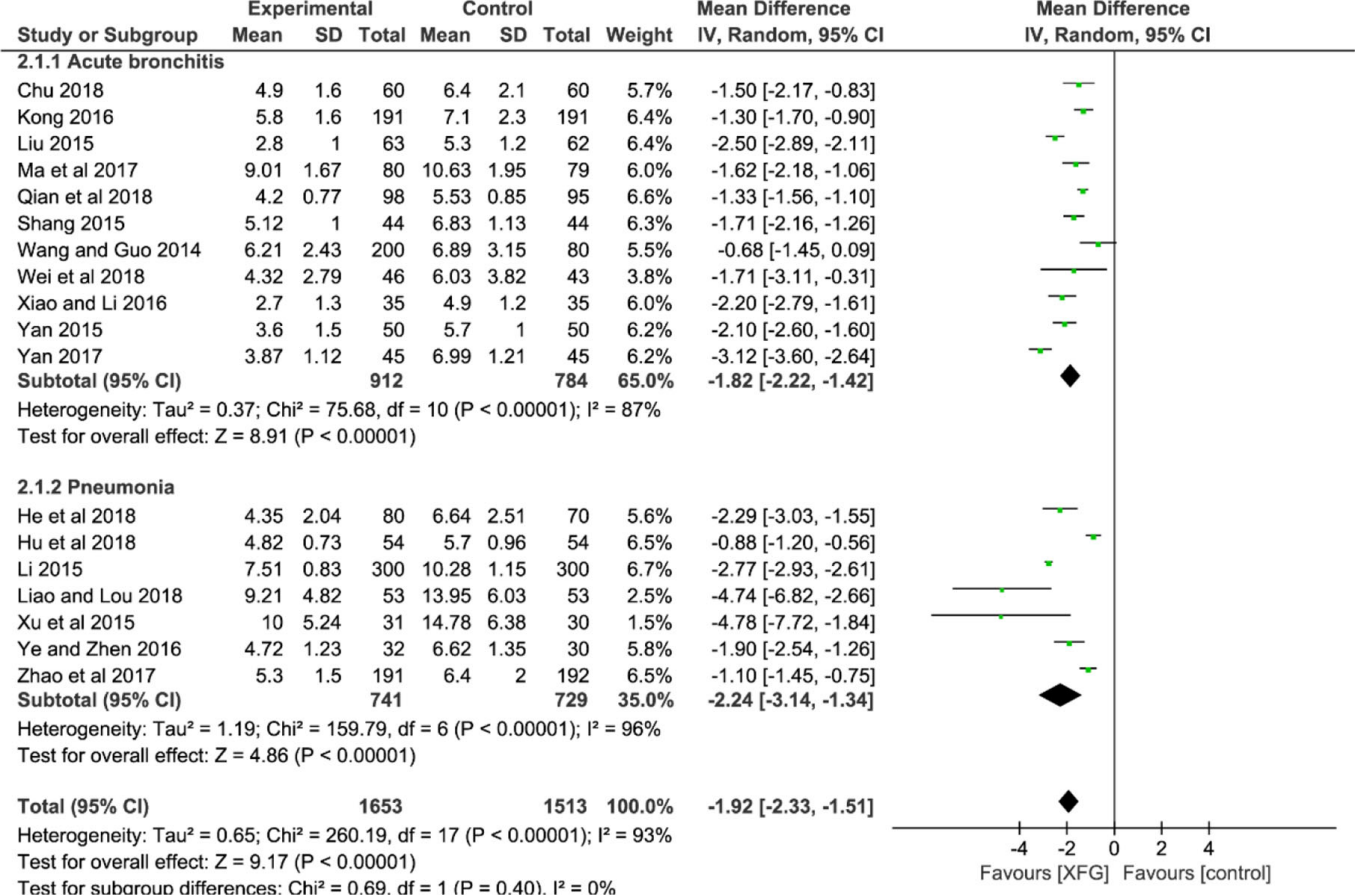
Forest plot and subgroup analysis of the RTC.

Eighteen studies used the RTC as an outcome after treatment (Wang and Guo, 2014; Li, 2015; Liu, 2015; Shang, 2015; Xu et al., 2015; Yan, 2015; Kong, 2016; Xiao and Li, 2016; Ye and Zhen, 2016; Ma and Lu, 2017; Yan, 2017; Zhao and Chen, 2017; Chu, 2018; He and Yang, 2018; Hu et al., 2018; Liao and Lou, 2018; Qian and Li, 2018; Wei and Li, 2018). An MD with a random-effects model was used to synthesize the data due to its striking heterogeneity (*P*<0.00001, *I^2^* = 93%). The result suggested that the RTC of the XFG group was reduced more effectively than that of the CM group (*MD* = -1.92; 95% *CI* =-2.33, -1.51; and *P*<0.00001). Additionally, there was no significant difference between the two subgroups (*p*=0.40, *I^2^* = 0%), as shown in **Figure 4**.

##### RTR

**Figure 5:**
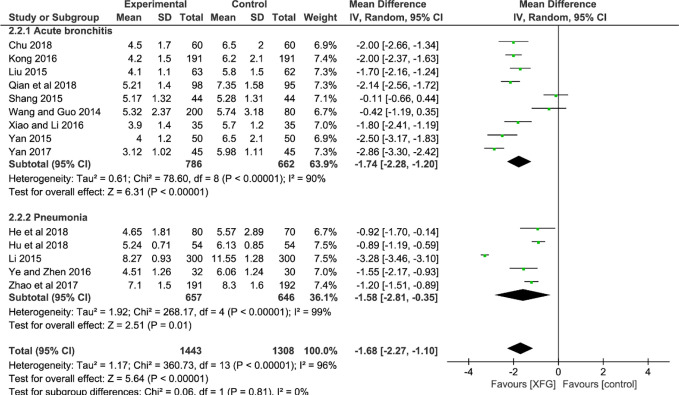
Forest plot and subgroup analysis of the RTR.

The RTR was reported in 14 studies (Wang and Guo, 2014; Li, 2015; Liu, 2015; Shang, 2015; Yan, 2015; Kong, 2016; Xiao and Li, 2016; Ye and Zhen, 2016; Yan, 2017; Zhao and Chen, 2017; Chu, 2018; He and Yang, 2018; Hu et al., 2018; Qian and Li, 2018). The result indicated a significant difference between the two groups with significant heterogeneity, suggesting that the RTR of the XFG group was shorter than that of the CM group (*MD* = -1.68, 95% *CI* =-2.27, -1.10, and *P*<0.00001). No significant difference was observed between the two subgroups (*P*=0.81, *I^2^* = 0%), as shown in **Figure 5**.

##### RTF

**Figure 6:**
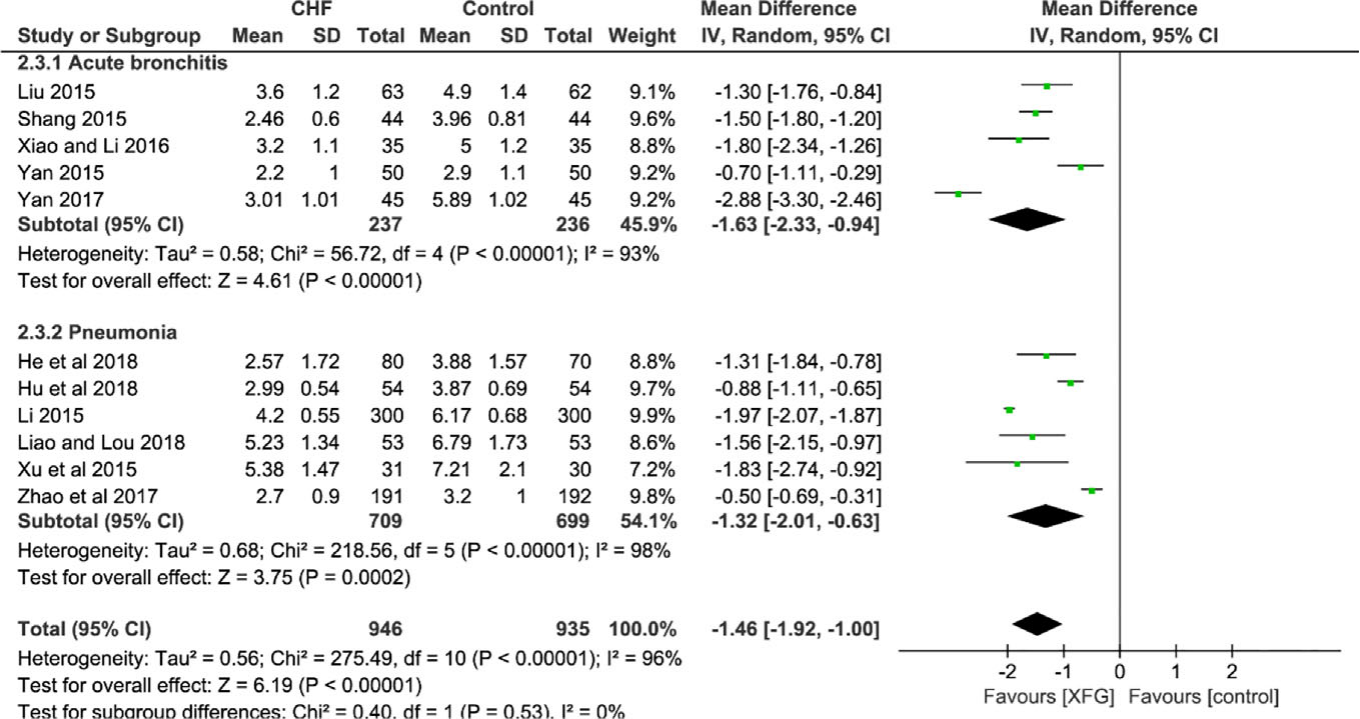
Forest plot and subgroup analysis of the RTF.

Eleven studies employed the outcome of the RTF (Liu, 2015; Shang, 2015; Yan, 2015; Li, 2015; Xu et al., 2015; Xiao and Li, 2016; Yan, 2017; Zhao and Chen, 2017; He and Yang, 2018; Hu et al., 2018; Liao and Lou, 2018). In a pooled analysis of the 11 trials, the addition of XFG led to a greater decrease in the RTF when compared with the CM therapy alone (*MD*=-1.46, 95% *CI* =-1.92, -1.00, and *P*< 0.00001), with high heterogeneity (*P*< 0.00001, *I^2^* = 96%), and there was no significant difference between the two subgroups (*P*=0.53, *I^2^* = 0%), as shown in **Figure 6**.

#### Secondary Outcomes

##### HS and RTIL

**Figure 7:**
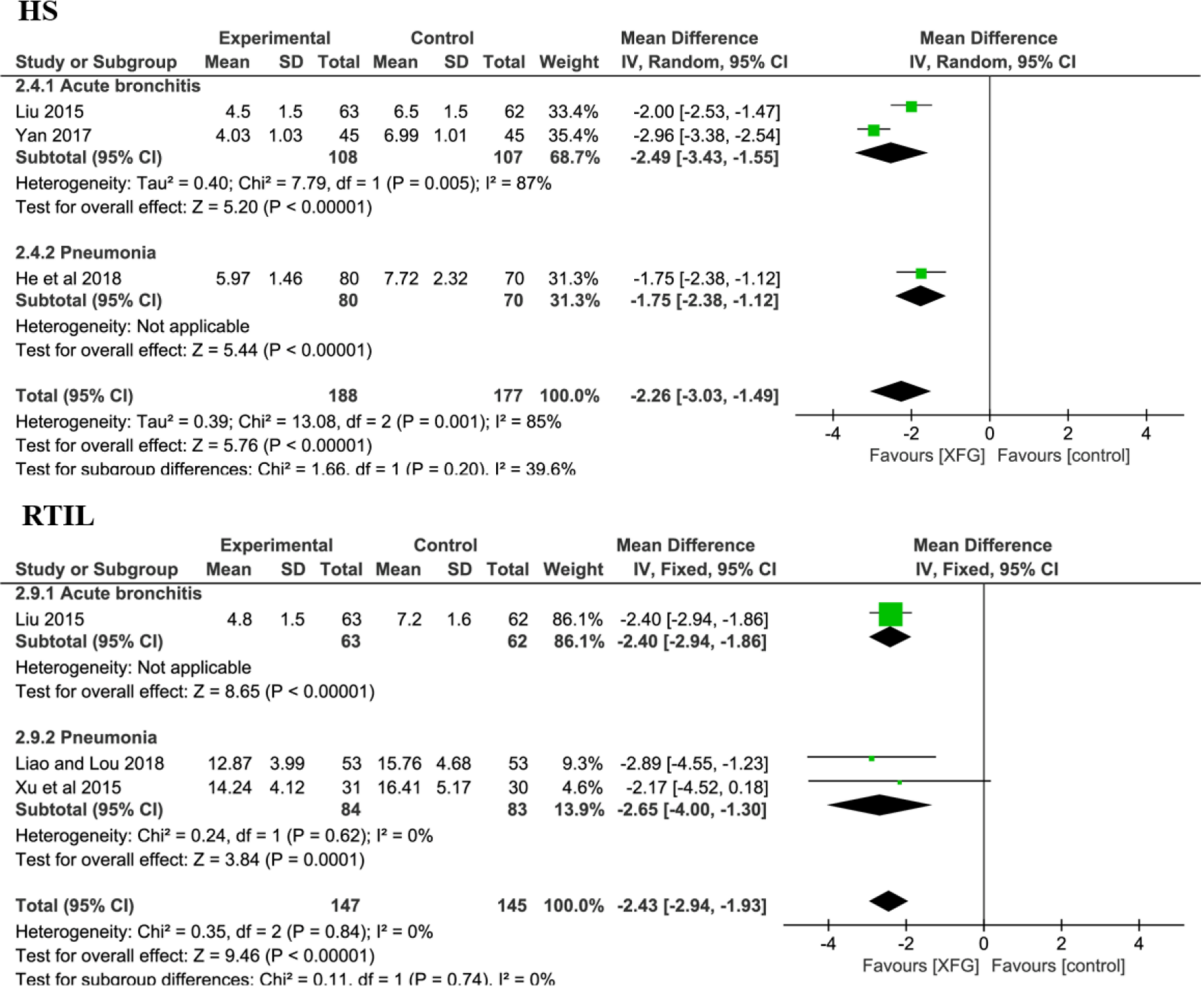
Forest plot and subgroup analysis of the HS and RTIL.

There were 3 RCTs (Liu, 2015; Yan, 2017; He and Yang, 2018) with a total of 365 patients on the HS (XFG group: 188 and CM group: 177). Compared with CM therapy alone, adding XFG to ALRI treatment significantly reduced the duration of the HS (*MD* = -2.26, 95% *CI* =-3.03, -1.49, *P*< 0.00001). Additionally, the RTIL was determined by X-ray in three studies involving 221 patients (Liu, 2015; Liao and Lou, 2018; Xu et al., 2015). The fixed-effects model was employed after a heterogeneity test (*P*=0.74; *I^2^* = 0%). Significantly, the meta-analysis results favored the XFG group, indicating that the XFG group was better than the CM group at accelerating the disappearance of inflammatory lesions (*MD* = -2.43, 95% *CI* =-2.94, -1.93, and *P*< 0.00001), as shown in **Figure 7**.

### Immune Cells and Cytokines

**Figure 8:**
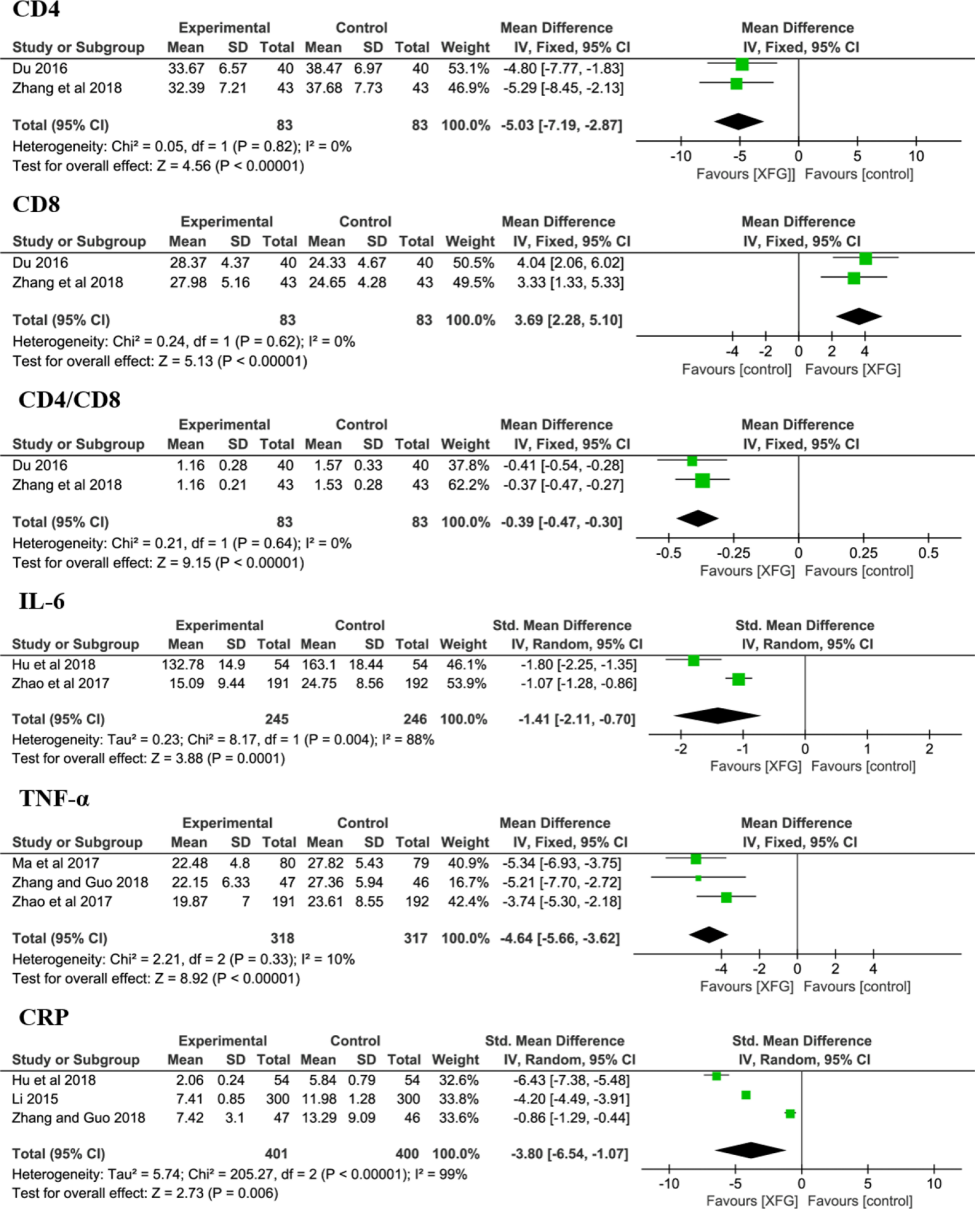
Forest plot and meta-analysis of CD4, CD8, CD4/CD8, IL-6, TNF-α, and CRP levels.

At the cellular and molecular levels, the immune cells (CD4 and CD8) and cytokines (IL-6, TNF-α and CRP levels) were determined in some trials (Li, 2015; Du, 2016; Zhao and Chen, 2017; Ma and Lu, 2017; Hu et al., 2018; Zhang and Guo, 2018; Zhang J. Q. et al., 2018). The results suggested that the levels of CD4 (*MD* = -5.03, 95% *CI* =-7.19, -2.83, *P*< 0.00001), CD8 (*MD* = 3.69, 95% *CI* =2.28, 5.10, *P*< 0.00001), CD4/CD8 (*MD* = -0.39, 95% *CI* =-0.47, -0.30, *P*< 0.00001), IL-6 (*sMD* = -1.41, 95% *CI* =-2.11, -0.70, *P*=0.0001), TNF-α (*MD* = -4.64, 95% *CI* =-5.66, -3.62, *P*< 0.00001) and CRP (*sMD* = -3.80, 95% *CI* =-6.54, -1.07, *P*=0.006) were significantly improved when complemented with XFG, indicating that adding XFG greatly reduced inflammation and enhanced the immune functions of the patient, as shown in **Figure 8**.

### Adverse Events

**Figure 9:**
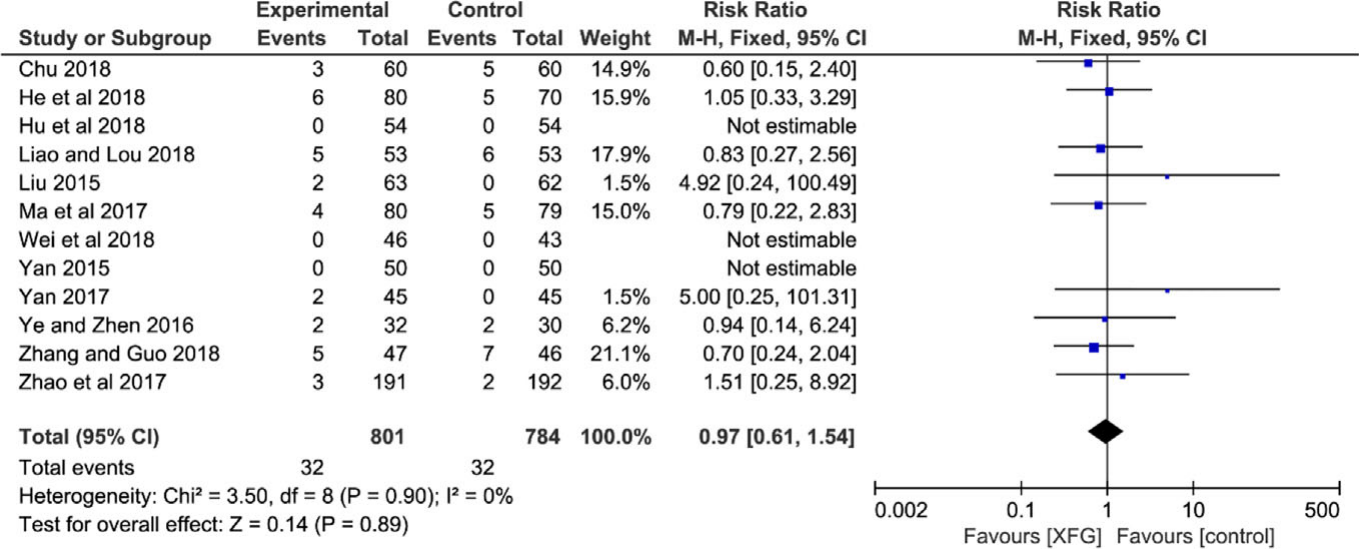
Forest plot and meta-analysis of adverse events.

Adverse events were mentioned in fifteen trials (Liu, 2015; Xu et al., 2015; Yan, 2015; Du, 2016; Ye and Zhen, 2016; Ma and Lu, 2017; Yan, 2017; Zhao and Chen, 2017; Chu, 2018; He and Yang, 2018; Hu et al., 2018; Liao and Lou, 2018; Wei and Li, 2018; Zhang J. Q. et al., 2018; Zhang and Guo, 2018). However, the remaining six trials did not record any adverse reaction information. Only three of the fifteen trials reported that there were no serious adverse reactions (Xu et al., 2015; Du, 2016; Zhang J. Q. et al., 2018). One of the trials reported mild gastrointestinal discomfort without statistics. Therefore, twelve studies detailed adequate information on adverse events (XFG: 32/801, 4.0%; CM: 32/784, 4.1%), and three reported no adverse events during trials (Yan, 2015; Hu et al., 2018; Wei and Li, 2018). The most frequent adverse reactions mentioned in these studies were gastrointestinal discomfort symptoms (nausea, diarrhea, and emesis) and dizziness, headache, and rash. In addition, one trial (Zhang and Guo, 2018) reported 3 cases with electrolyte disturbance (XFG: 1/47, 2.1%; CM: 2/46, 4.3%). In general, all the adverse reactions were mild, and no serious adverse reactions were reported. The pooled result indicated no statistical difference between the two groups, suggesting that adding XFG did not increase the adverse events (*RR* =0.97, 95% *CI=* 0.61-1.54, and *P=* 0.89), as shown in **Figure 9**.

### Subgroup Analysis

Subgroup analyses of the treatment durations were introduced to determine the subgroup differences according to the primary outcomes, including the CER, RTC, RTR, and RTF. For the CER, RTC, and RTR, the results across the various subgroups were highly consistent, and the benefits of XFG were significant. However, a significant difference was observed between the “5-8 days” and “14 days” groups in terms of the RTF. Generally, the “14 day” group showed a lower effect size value and a narrower confidence interval than that of the “5-8 days” group, which indicated that “14 days” of treatment was more likely to bring the maximum therapeutic effect to the ALRI patients in terms of improving the RTF. Despite the significant difference favoring the 14-day group, it is unreasonable to conclude that 14 days of XFG was more effective than 5-8 days because the number of studies between subgroups varied widely, and there are some differences between bronchitis and pneumonia. In terms of the dose, most studies followed the packaging instructions for the drug provided by the two pharmaceutical companies (ages: 0-1 (2g); 1-4 (3g); 5-8 (6g), tid, PO). Therefore, considering the large age difference between the studies and the ALRI severity difference, it is reasonable to adjust the medication according to different conditions, as shown in **Table 2**.

**Table 2:** Subgroup analysis for treatment duration.

Subgroups	Studies	No. ofParticipants (n_E_/n_C_)	Effects model	Pooled effect	95% Cl	P value
CER						
Treatment duration (5-8 days)	14	1180/1044	Fixed	RR 1.16	1.16, 1.21	<0.00001
Treatment duration (14 days)	7	603/598	Fixed	RR 1.20	1.09, 1.31	<0.00001
Total	21	1783/1642	Fixed	RR 1.17	1.13, 1.22	<0.00001
Test for subgroup differences: Chi^2^ = 0.31, df=1 (P=0.58), I^2^ = 0%						
RTC						
Treatment duration (5-8 days)	12	1097/961	Random	MD -1.74	-2.14, -1.33	<0.00001
Treatment duration (14 days)	6	556/552	Random	MD -2.35	-3.09, -1.61	<0.00001
Total	18	1653/1513	Random	MD -1.92	-2.23, -1.51	<0.00001
Test for subgroup differences: Chi^2^ = 2.03, df=1 (P=0.15), I^2^ = 50.7%						
RTR						
Treatment duration (5-8 days)	11	1051/918	Random	MD -1.52	-1.99, -1.05	<0.00001
Treatment duration (14 days)	3	392/390	Random	MD -2.31	-3.52, -1.09	0.0002
Total	14	1443/1308	Random	MD -1.68	-2.27. -1.10	<0.00001
Test for subgroup differences: Chi^2^ = 1.40, df=1 (P=0.24), I^2^ = 28.5%						
RTF						
Treatment duration (5-8 days)	8	556/552	Random	MD -1.35	-1.85, -0.84	<0.00001
Treatment duration (14 days)	3	384/383	Random	MD -1.96	-2.05, -1.86	<0.00001
Total	11	946/935	Random	MD -1.46	-1.92, -1.00	<0.00001

Test for subgroup differences: Chi^2^ = 5.40, df=1 (P=0.02), I^2^ = 81.5%.

### Sensitivity Analysis

**Figure 10:**
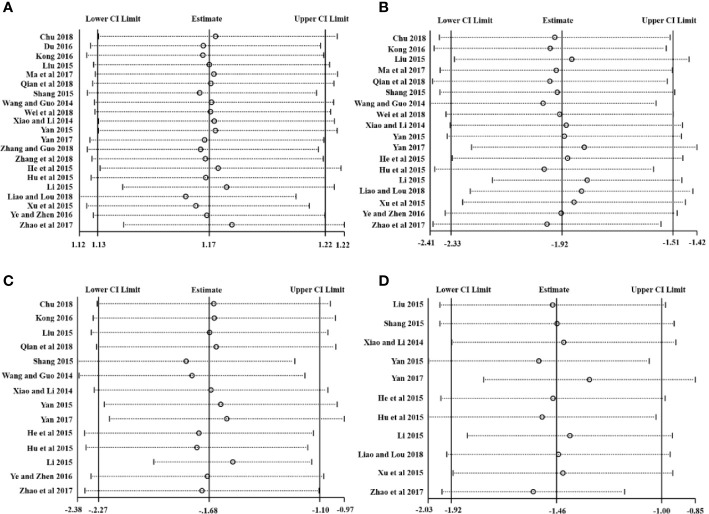
Sensitivity analysis plots of **(A)** CER, **(B)** RTC, **(C)** RTR, and **(D)** RTF.

Stata 15.1 was employed for sensitivity analysis of the primary outcomes including the CEF, RTC, RTR, and RTF. The centralized small circles in the sensitivity analysis plots suggested that the results were not reversed by removing any study for each outcome, indicating that the results were reliable, as shown in **Figure 10**.

### Publication Bias

**Figure 11:**
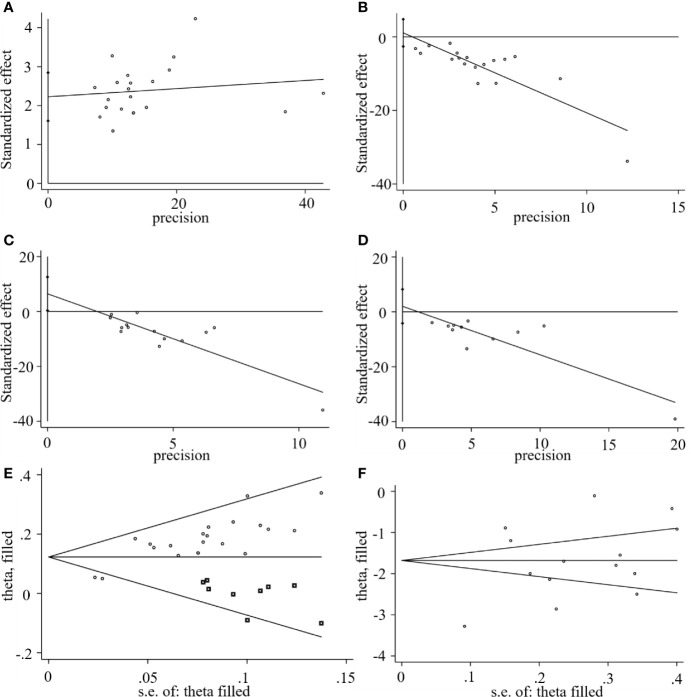
Egger’s publication bias plots of the **(A)** CER, **(B)** RCT, **(C)** RTR, **(D)** RTF, and filled funnel plots of the **(E)** CER, and **(F)** RTR.

We used Stata15.1 software to detect possible publication bias according to primary outcomes, and the trim and filling method was used to cope with the striking publication bias if the *P*<0.05. The result of Egger’s test suggested that there was no publication bias in terms of the RTC (*P* >| *t* |=0.542, 95% *CI=* −2.6 to 4.79) and RTF (*P* >| *t* |=0.48, 95% *CI=* -4.17 to 8.21). However, significant publication bias was observed in the CER (*P* >| *t* |=0.0001, 95% *CI=* 1.61-2.84) and the RTR (*P* >| *t* |=0.04, 95% *CI=* 0.35 to 12.56). The trim and filling method was then used to evaluate the reliability of results affected by significant publication bias. After two iterations, no potential missing study was filled. The result suggested that the *MD* and 95% *CI* of the RTR (*MD* = -1.68; 95% *CI*=-2.26 to -1.0; *P*<0.0001) vs. (*MD* = -1.68; 95% *CI*=-2.26 to -1.0; *P*<0.0001) before and after applying the trim and filling method was consistent, indicating that the result was stable without a flip. For the CER, after four iterations, nine studies marked with squares were filled in. The results showed that the *RR* and 95% *CI* (*RR*=1.17, 95% *CI* =1.13-1.22, *P*< 0.00001) versus (*RR* = 1.13; 95% *CI*=1.09-1.17; *P*<0.0001) before and after applying the trim and filling method was consistent. Generally, the publication bias induced by CER and RTR had no significant effect on the pooled results, as shown in **Figure 11**.

## Discussion

This meta-analysis assessed the evidence from 21 RCTs. A total of 3425 ALRI patients were randomly assigned to receive either an XFG addition with CM or CM alone. The findings of this study suggested that XFG supplementation of the ALRI treatment was effective and safe, and it was associated with significant improvements in the RTC, RTR, RTF, RTIL, CD4, CD8, IL-6, TNF-α, and CRP. Both the symptoms and immunity-related outcomes of the patients were significantly improved when CM was complemented with XFG, which indirectly reduced the use of antibiotics. However, substantial heterogeneity was present in the three primary outcomes (RTC, RTR, and RTF). According to the sensitivity analysis, two studies (Li, 2015; Yan, 2017) significantly increased the overall heterogeneity. A possible explanation for this finding is that Li, 2015 had a larger sample size compared with the other 20 included studies, while Yan, 2017 had a small sample size. In addition, differences in age and the severity of the disease appear to be another important source of heterogeneity.

**Figure 12:**
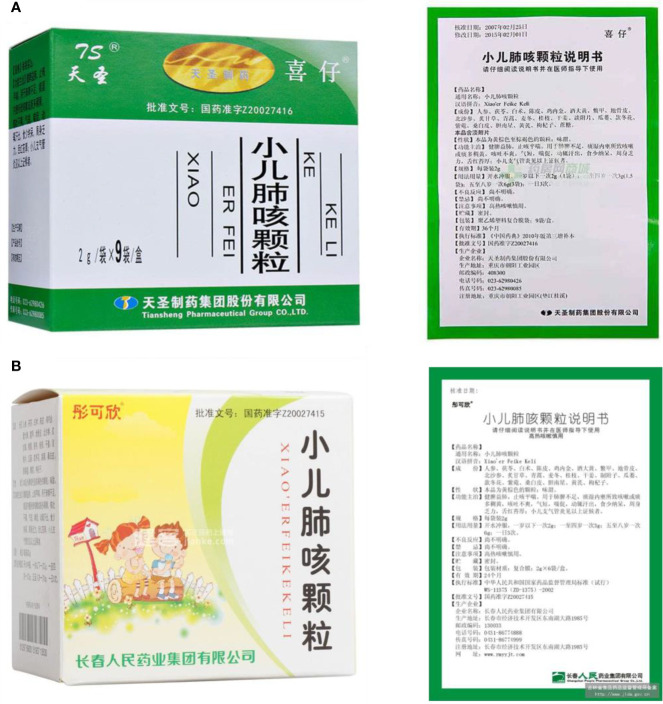
XFG and its instructions from **(A)** Tian-sheng Pharmaceutical Group Co., LTD, Chong-qing, China, and **(B)** Chang-chun People Pharmaceutical Group Co., LTD, Chang-chun, China.

XFG is a patented preparation. Two Chinese pharmaceutical companies, Tian-sheng Pharmaceutical Group Co., LTD, and Chang-chun People Pharmaceutical Group Co., LTD, produce XFG according to the guidelines outlined in the latest edition of the Chinese pharmacopoeia, which consists of Ginseng radix et rhizoma, Poria, *Atractylodis macrocephalae* rhizoma, Citri reticulatae pericarpium, Rhei radix et rhizome, Lycii cortex, Glehniae radix, Glycyrrhizae radix et rhizome, *Artemisiae annuae* herba, Ophiopogonis radix, Cinnamomic ramulus, Zingiberis rhizoma, Aconiti radix, Trichosanthis fructus, Farfarae flos, Asteris radix et rhizome, Mori cortex, Astragali radix, Lycii fructu, Arisaema cum Bile, Galli gigerii endothelium corneum, Trionycis carapax, and saccharose. The procedure for making this preparation is as follows. First, ten herbs are mixed, including Astragali radix, Lycii cortex, Glehniae radix, Glehniae radix, Glycyrrhizae radix et rhizome, Artemisiae annuae herba, Cinnamomic ramulus, Trichosanthis fructus, Asteris radix et rhizome, and Mori cortex. They are decocted with water twice, for 2 hours per decoction, and the decoctions are combined, filtered, and concentrated to form a thin extract with a relative density of 1.26-1.30 (80°C). Second, the other twelve ingredients are pulverized into a powder. Third, in addition to the above thin extract, the powder and saccharose are mixed, dried, and made to reach 1000g. This preparation method for XFG is stable and repeatable (**Figure 12**).

The primary pharmacological effects of the 22 ingredients include lung injury protection, enhancing immune function, anti-microbial, anti-inflammatory, and anti-asthmatic activities, neuroprotection, and antitussive and expectorant effects. The majority of the 22 ingredients have strong anti-microbial and anti-inflammatory properties, which significantly enhanced resistance to pathogenic microorganisms in ALRI patients. Seven herbs, namely, Citri reticulatae pericarpium, Glycyrrhizae radix et rhizome, Glycyrrhizae radix et rhizome, Trichosanthis fructus, Farfarae flos, Trichosanthis fructus, and Mori cortex, are widely used for antitussive, expectorant, and anti-asthmatic effects by TCM physicians for thousands of years. Modern pharmacology has also confirmed these effects on respiratory disease (Ruan and Yue, 2004; Shi et al., 2009; Sun et al., 2012; Wang et al., 2014; Kim et al., 2017; Singh et al., 2018; Fu et al., 2019; Li et al., 2019). Seven ingredients (Ginseng radix et rhizoma, Poria, *Atractylodis macrocephalae* rhizoma, Ophiopogonis radix, Astragali radix, Astragali radix, and Trionycis carapax) showed biological activity in immune enhancement, especially Ginseng radix et rhizome, which significantly increased the viability and proliferation of spleen cells by promoting proliferation from CD19(+) B cells and increased the surface expression of CD25, CD69, and interleukin-2, which significantly enhanced the immune system of ALRI patients. Two herbs (Lycii cortex and Cinnamomic ramulus) showed striking neuroprotection effects, with kukoamine A (Zhang et al., 2016) from Cortex lycii reducing the malondialdehyde level and increasing the glutathione level, superoxide dismutase, catalase (CAT) activities, and the expression of BDNF. Three herbs significantly improved acute lung injury in mice (Asteris radix et rhizome, Astragali radix, and Lycii fructus) due to mechanisms that enhanced the activation of Nrf2, inducing human cathelicidin antimicrobial peptide LL-37 in respiratory epithelial cells, promoting the repair of vascular endothelial cells, and inhibiting NF-κB signaling pathway. On the whole, the TCM efficacy of XFG was consistent with its pharmacological effects, and XFG plays a therapeutic role through coordinated cooperation among the 22 ingredients. Therefore, based on TCM theory, through a multiple-target pathway, multi-level and holistic therapy, XFG was found to be a reliably effective and safe complementary therapy in ALRI treatment, as shown in **Tables 3**, **4**.

**Table 3:** Applications of the 22 ingredients of XFG in traditional Chinese medicine.

Chinese names of ingredients	Primary TCM efficacy (Zhang, 2018)	Effective chemical components	Pharmacological effects
Ginseng radix et rhizoma	Tonifying Qi	Ginsenosides Panaxan	Enhance immune function (Ko and Joo, 2010; Wang et al., 2013) Anti-microbial (Kim and Yang, 2018). Anti-inflammatory (Park et al., 2004; Jung et al., 2010)
Poria	Inducing diuresis to alleviate edema	Pachymaran Pachymic acid	Enhance immune function (Yu and Tseng, 1996) Anti-inflammatory (Giner et al., 2000; Prieto et al., 2003)
Atractylodis macrocephalae rhizoma	Qi-tonifying and spleen fortifying	Atractylon, Atractylol, Atractylenolide	Enhance immune function (Li et al., 2009; Guo et al., 2012) Anti-microbial (Cheng et al., 2016) Anti-inflammatory (Bose and Kim, 2013; Zhang et al., 2015)
Citri reticulatae Pericarpium	Qi-regulating and spleen- fortifying, drying dampness and resolving phlegm	Volatile oil, Hesperidin, Nobiletin, Naringin	Anti-asthmatic activity (Shi et al., 2009; Fu et al., 2019) Anti-microbial (Casquete et al., 2015) Anti-inflammatory (Ha et al., 2012; Hagenlocher et al., 2016),
Rhei radix et rhizome	Removing accumulation with purgation heat-clearing, fire-purging and removing toxicity	Anthraquinones	Anti-microbial (Smolarz et al., 2013; Arokiyaraj et al., 2017; Khiveh et al., 2017; Keser et al., 2019)
Lycii cortex	Clearing deficiency-heat and cooling blood	Kukoamine A, Kukoamine B Lyciumin A, Lyciumin B	Neuroprotection (Hu et al., 2015; Zhang et al., 2016). Anti-inflammatory (Hadjipavlou-Litina et al., 2009; Zhang J. et al., 2014) Anti-microbial (Han et al., 2002; Lee et al., 2004)
Glehniae radix	Nourishing yin and lung	Polysaccharide, Coumarins	Enhance immune function (Liu et al., 2005) Anti-inflammatory (Huang et al., 2012)
Glycyrrhizae radix et rhizome	Resolving phlegm and relieving cough	Glycyrrhizins, Flavonoids	Anti-asthmatic activity (Kim et al., 2017)
Artemisiae annuae herba	Clearing deficiency-heat and cooling blood	Artemisinin Volatile oil	Anti-inflammatory; Anti-microbial, Anti-malarial (Charlie-Silva and Fraceto, 2018; Efferth, 2018)
Ophiopogonis radix	Nourishing yin and moistening lung	Ophiopogonins	Enhance immune function (Lu et al., 2017), Anti-inflammatory (Guo et al., 2019)
Cinnamomic ramulus	Promoting sweating to releasing the flesh	Cinnamaldehyde	Anti-malarial (Friedman, 2017; Albano et al., 2019) Neuroprotection (Zheng et al., 2015)
Zingiberis rhizoma	Warming the middle and dispersing cold	Volatile oil Gingerol	Anti-asthmatic activity (Singh et al., 2018; Li et al., 2019) Anti-microbial (Lee et al., 2018)
Aconiti radix	Reviving yang for resuscitation	Aconitines	Anti-microbial (Xu W. et al., 2017)
Trichosanthis fructus	Clearing heat and washing away the phlegm	Tetradecanoic acid Fumaric acid, Succinic acid	Anti-asthmatic activity (Huang and Yao, 1984) Antitussive and expectorant effects (Ruan and Yue, 2004; Sun et al., 2012)
Farfarae flos	Resolving phlegm and relieving cough	Flavonoids, Tussilagone, Alkaloids	Antitussive and expectorant effects (Cheng et al., 2018; Li et al., 2018a; Li et al., 2018b)
Asteris radix et rhizome	Resolving phlegm and relieving cough	Shionones, Flavonoids Astersaponin	Antitussive and expectorant effects (Yang et al., 2008; Liu et al., 2011; Yu et al., 2015) Lung-injury protection (Chen et al., 2019)
Mori cortex	Purging the lung to calm panting Inducing diuresis to alleviate edema	Mul-berin, Morusin, Cy-omorusin	Antitussive and expectorant effects (Wang et al., 2014) Anti-asthmatic activity (Kim et al., 2011; Sui et al., 2015)
Astragali radix	Qi-tonifying and yang- lifting	Astragaloside, Astragalus polysaccharide	Enhance immune function (Li et al., 2017; Liu et al., 2018) Lung-injury protection (Wang and Huang, 2014; Su et al., 2015; Qin et al., 2018; Zhao et al., 2018)
Lycii fructus	Nourishing liver and kidney	Lycium barbarum polysaccharide	Enhance immune function (Zhang X. et al., 2014; Zhang et al., 2019) Lung-injury protection (Chen et al., 2018; Zheng et al., 2019)
Arisaema cum Bile	Clearing heat and resolving phlegm	Bile acids	Defervescence (Chen et al., 2017) Anti-inflammatory (Ahn and Je, 2012)
Galli gigerii endothelium corneum	Removing stagnated food by regulating stomach	Gastric hormone, Pepsase, amylase	Improve digestion (Li et al., 2008; Xu X. P. et al., 2017)
Trionycis carapax	Nourishing and suppressing Yang; heat-clearing	Ossein, keratin Amino acids	Enhance immune function (Wang and Guan, 2010; Zhang et al., 2004)

**Table 4:** The general characters of XFG.

Study	Formulation, Source, Species, concentration	Quality control reported? (Y/N)	Chemical analysis reported? (Y/N)
Chu, 2018	**XFG, [Chang-chun people pharmaceutical group co., Ltd]**. (1)Dried roots of Panax ginseng C. A. Mey., 20g; (2)Poria cocos (Schw) Wolf., 20g; (3)Dried rhizomes of Atractylodes macrocephala Koidz., 8g; (4)Aged fruit peel of Citrus reticulata Blanco., 20g., (5) Dried rhizomes of Rheum palmatum L., 12g; (6) Dried root peels of Lycium chinense Mill., 23g., (7) Dried roots of Glehnia littoralis Fr. Schmidtex Miq., 39g; (8) Dried rhizomes of Glycyrrhiza uralensis Fisch., 12g; (9)Artemisia annua L., 29g; (10) Dried roots of Ophiopogon japonicus (L.f) Ker-Gawl., 39g; (11) Dried twigs of Cinnamomum cassia Presl., 8g; (12) Dried rhizomes of Zingiber officinale Rose., 8g; (13) Dried roots of Aconitum carmichaelii Debx., 8g; (14)Dried fruits of Trichmanthes kiriloxvii Maxim., 29g; (15)Dried flower buds of Tussilago farfara L., 20g; (16) Dried rhizomes of Aster tataricus L. f., 20g; (17) Dried root peels of Morus alba L., 23g; (18) Dried roots of Astragalus membranaceus (Fisch.)Bge., 20g; (19)Dried fruits of Lycium barbarum Mill., 20g; (20) Dried root powder of Arisaema erubescens (Wall.) Schott with (ox bile or sheep bile or pig bile)., 8g; (21) Dried stomach lining of Callus gallus domesticus Brisson., 20g; (22) Dried turtle shell of Trionyx sinensis Wiegmann., 20g	Y-Prepared according to Chinese pharmacopoeia	Y - HPLC
Du, 2016	**XFG, [Tian-sheng pharmaceutical group co., Ltd]**. (1)Dried roots of Panax ginseng C. A. Mey., 20g; (2)Poria cocos (Schw) Wolf., 20g; (3)Dried rhizomes of Atractylodes macrocephala Koidz., 8g; (4)Aged fruit peel of Citrus reticulata Blanco., 20g., (5) Dried rhizomes of Rheum palmatum L., 12g; (6) Dried root peels of Lycium chinense Mill., 23g., (7) Dried roots of Glehnia littoralis Fr. Schmidtex Miq., 39g; (8) Dried rhizomes of Glycyrrhiza uralensis Fisch., 12g; (9)Artemisia annua L., 29g; (10) Dried roots of Ophiopogon japonicus (L.f) Ker-Gawl., 39g; (11) Dried twigs of Cinnamomum cassia Presl., 8g; (12) Dried rhizomes of Zingiber officinale Rose., 8g; (13) Dried roots of Aconitum carmichaelii Debx., 8g; (14)Dried fruits of Trichmanthes kiriloxvii Maxim., 29g; (15)Dried flower buds of Tussilago farfara L., 20g; (16) Dried rhizomes of Aster tataricus L. f., 20g; (17) Dried root peels of Morus alba L., 23g; (18) Dried roots of Astragalus membranaceus (Fisch.)Bge., 20g; (19)Dried fruits of Lycium barbarum Mill., 20g; (20) Dried root powder of Arisaema erubescens (Wall.) Schott with (ox bile or sheep bile or pig bile)., 8g; (21) Dried stomach lining of Callus gallus domesticus Brisson., 20g; (22) Dried turtle shell of Trionyx sinensis Wiegmann., 20g	Y-Prepared according to Chinese pharmacopoeia	Y - HPLC
Kong, 2016	**XFG, [Tian-sheng pharmaceutical group co., Ltd]**. (1)Dried roots of Panax ginseng C. A. Mey., 20g; (2)Poria cocos (Schw) Wolf., 20g; (3)Dried rhizomes of Atractylodes macrocephala Koidz., 8g; (4)Aged fruit peel of Citrus reticulata Blanco., 20g., (5) Dried rhizomes of Rheum palmatum L., 12g; (6) Dried root peels of Lycium chinense Mill., 23g., (7) Dried roots of Glehnia littoralis Fr. Schmidtex Miq., 39g; (8) Dried rhizomes of Glycyrrhiza uralensis Fisch., 12g; (9)Artemisia annua L., 29g; (10) Dried roots of Ophiopogon japonicus (L.f) Ker-Gawl., 39g; (11) Dried twigs of Cinnamomum cassia Presl., 8g; (12) Dried rhizomes of Zingiber officinale Rose., 8g; (13) Dried roots of Aconitum carmichaelii Debx., 8g; (14)Dried fruits of Trichmanthes kiriloxvii Maxim., 29g; (15)Dried flower buds of Tussilago farfara L., 20g; (16) Dried rhizomes of Aster tataricus L. f., 20g; (17) Dried root peels of Morus alba L., 23g; (18) Dried roots of Astragalus membranaceus (Fisch.)Bge., 20g; (19)Dried fruits of Lycium barbarum Mill., 20g; (20) Dried root powder of Arisaema erubescens (Wall.) Schott with (ox bile or sheep bile or pig bile)., 8g; (21) Dried stomach lining of Callus gallus domesticus Brisson., 20g; (22) Dried turtle shell of Trionyx sinensis Wiegmann., 20g	Y-Prepared according to Chinese pharmacopoeia	Y - HPLC
Liu, 2015	**XFG, [Tian-sheng pharmaceutical group co., Ltd]**. (1)Dried roots of Panax ginseng C. A. Mey., 20g; (2)Poria cocos (Schw) Wolf., 20g; (3)Dried rhizomes of Atractylodes macrocephala Koidz., 8g; (4)Aged fruit peel of Citrus reticulata Blanco., 20g., (5) Dried rhizomes of Rheum palmatum L., 12g; (6) Dried root peels of Lycium chinense Mill., 23g., (7) Dried roots of Glehnia littoralis Fr. Schmidtex Miq., 39g; (8) Dried rhizomes of Glycyrrhiza uralensis Fisch., 12g; (9)Artemisia annua L., 29g; (10) Dried roots of Ophiopogon japonicus (L.f) Ker-Gawl., 39g; (11) Dried twigs of Cinnamomum cassia Presl., 8g; (12) Dried rhizomes of Zingiber officinale Rose., 8g; (13) Dried roots of Aconitum carmichaelii Debx., 8g; (14)Dried fruits of Trichmanthes kiriloxvii Maxim., 29g; (15)Dried flower buds of Tussilago farfara L., 20g; (16) Dried rhizomes of Aster tataricus L. f., 20g; (17) Dried root peels of Morus alba L., 23g; (18) Dried roots of Astragalus membranaceus (Fisch.)Bge., 20g; (19)Dried fruits of Lycium barbarum Mill., 20g; (20) Dried root powder of Arisaema erubescens (Wall.) Schott with (ox bile or sheep bile or pig bile)., 8g; (21) Dried stomach lining of Callus gallus domesticus Brisson., 20g; (22) Dried turtle shell of Trionyx sinensis Wiegmann., 20g	Y-Prepared according to Chinese pharmacopoeia	Y - HPLC
Ma and Lu, 2017	**XFG, [Tian-sheng pharmaceutical group co., Ltd]**. (1)Dried roots of Panax ginseng C. A. Mey., 20g; (2)Poria cocos (Schw) Wolf., 20g; (3)Dried rhizomes of Atractylodes macrocephala Koidz., 8g; (4)Aged fruit peel of Citrus reticulata Blanco., 20g., (5) Dried rhizomes of Rheum palmatum L., 12g; (6) Dried root peels of Lycium chinense Mill., 23g., (7) Dried roots of Glehnia littoralis Fr. Schmidtex Miq., 39g; (8) Dried rhizomes of Glycyrrhiza uralensis Fisch., 12g; (9)Artemisia annua L., 29g; (10) Dried roots of Ophiopogon japonicus (L.f) Ker-Gawl., 39g; (11) Dried twigs of Cinnamomum cassia Presl., 8g; (12) Dried rhizomes of Zingiber officinale Rose., 8g; (13) Dried roots of Aconitum carmichaelii Debx., 8g; (14)Dried fruits of Trichmanthes kiriloxvii Maxim., 29g; (15)Dried flower buds of Tussilago farfara L., 20g; (16) Dried rhizomes of Aster tataricus L. f., 20g; (17) Dried root peels of Morus alba L., 23g; (18) Dried roots of Astragalus membranaceus (Fisch.)Bge., 20g; (19)Dried fruits of Lycium barbarum Mill., 20g; (20) Dried root powder of Arisaema erubescens (Wall.) Schott with (ox bile or sheep bile or pig bile)., 8g; (21) Dried stomach lining of Callus gallus domesticus Brisson., 20g; (22) Dried turtle shell of Trionyx sinensis Wiegmann., 20g	Y-Prepared according to Chinese pharmacopoeia	Y - HPLC
Qian and Li, 2018	**XFG, [Chang-chun people pharmaceutical group co., Ltd]**. (1)Dried roots of Panax ginseng C. A. Mey., 20g; (2)Poria cocos (Schw) Wolf., 20g; (3)Dried rhizomes of Atractylodes macrocephala Koidz., 8g; (4)Aged fruit peel of Citrus reticulata Blanco., 20g., (5) Dried rhizomes of Rheum palmatum L., 12g; (6) Dried root peels of Lycium chinense Mill., 23g., (7) Dried roots of Glehnia littoralis Fr. Schmidtex Miq., 39g; (8) Dried rhizomes of Glycyrrhiza uralensis Fisch., 12g; (9)Artemisia annua L., 29g; (10) Dried roots of Ophiopogon japonicus (L.f) Ker-Gawl., 39g; (11) Dried twigs of Cinnamomum cassia Presl., 8g; (12) Dried rhizomes of Zingiber officinale Rose., 8g; (13) Dried roots of Aconitum carmichaelii Debx., 8g; (14)Dried fruits of Trichmanthes kiriloxvii Maxim., 29g; (15)Dried flower buds of Tussilago farfara L., 20g; (16) Dried rhizomes of Aster tataricus L. f., 20g; (17) Dried root peels of Morus alba L., 23g; (18) Dried roots of Astragalus membranaceus (Fisch.)Bge., 20g; (19)Dried fruits of Lycium barbarum Mill., 20g; (20) Dried root powder of Arisaema erubescens (Wall.) Schott with (ox bile or sheep bile or pig bile)., 8g; (21) Dried stomach lining of Callus gallus domesticus Brisson., 20g; (22) Dried turtle shell of Trionyx sinensis Wiegmann., 20g	Y-Prepared according to Chinese pharmacopoeia	Y - HPLC
Shang, 2015	**XFG, [Tian-sheng pharmaceutical group co., Ltd or Chang-chun people pharmaceutical group co., Ltd]**. (1)Dried roots of Panax ginseng C. A. Mey., 20g; (2)Poria cocos (Schw) Wolf., 20g; (3)Dried rhizomes of Atractylodes macrocephala Koidz., 8g; (4)Aged fruit peel of Citrus reticulata Blanco., 20g., (5) Dried rhizomes of Rheum palmatum L., 12g; (6) Dried root peels of Lycium chinense Mill., 23g., (7) Dried roots of Glehnia littoralis Fr. Schmidtex Miq., 39g; (8) Dried rhizomes of Glycyrrhiza uralensis Fisch., 12g; (9)Artemisia annua L., 29g; (10) Dried roots of Ophiopogon japonicus (L.f) Ker-Gawl., 39g; (11) Dried twigs of Cinnamomum cassia Presl., 8g; (12) Dried rhizomes of Zingiber officinale Rose., 8g; (13) Dried roots of Aconitum carmichaelii Debx., 8g; (14)Dried fruits of Trichmanthes kiriloxvii Maxim., 29g; (15)Dried flower buds of Tussilago farfara L., 20g; (16) Dried rhizomes of Aster tataricus L. f., 20g; (17) Dried root peels of Morus alba L., 23g; (18) Dried roots of Astragalus membranaceus (Fisch.)Bge., 20g; (19)Dried fruits of Lycium barbarum Mill., 20g; (20) Dried root powder of Arisaema erubescens (Wall.) Schott with (ox bile or sheep bile or pig bile)., 8g; (21) Dried stomach lining of Callus gallus domesticus Brisson., 20g; (22) Dried turtle shell of Trionyx sinensis Wiegmann., 20g	Y-Prepared according to Chinese pharmacopoeia	Y - HPLC
Wei and Li, 2018	**XFG, [Tian-sheng pharmaceutical group co., Ltd]**. (1)Dried roots of Panax ginseng C. A. Mey., 20g; (2)Poria cocos (Schw) Wolf., 20g; (3)Dried rhizomes of Atractylodes macrocephala Koidz., 8g; (4)Aged fruit peel of Citrus reticulata Blanco., 20g., (5) Dried rhizomes of Rheum palmatum L., 12g; (6) Dried root peels of Lycium chinense Mill., 23g., (7) Dried roots of Glehnia littoralis Fr. Schmidtex Miq., 39g; (8) Dried rhizomes of Glycyrrhiza uralensis Fisch., 12g; (9)Artemisia annua L., 29g; (10) Dried roots of Ophiopogon japonicus (L.f) Ker-Gawl., 39g; (11) Dried twigs of Cinnamomum cassia Presl., 8g; (12) Dried rhizomes of Zingiber officinale Rose., 8g; (13) Dried roots of Aconitum carmichaelii Debx., 8g; (14)Dried fruits of Trichmanthes kiriloxvii Maxim., 29g; (15)Dried flower buds of Tussilago farfara L., 20g; (16) Dried rhizomes of Aster tataricus L. f., 20g; (17) Dried root peels of Morus alba L., 23g; (18) Dried roots of Astragalus membranaceus (Fisch.)Bge., 20g; (19)Dried fruits of Lycium barbarum Mill., 20g; (20) Dried root powder of Arisaema erubescens (Wall.) Schott with (ox bile or sheep bile or pig bile)., 8g; (21) Dried stomach lining of Callus gallus domesticus Brisson., 20g; (22) Dried turtle shell of Trionyx sinensis Wiegmann., 20g	Y-Prepared according to Chinese pharmacopoeia	Y - HPLC
Wang and Guo, 2014	**XFG, [Chang-chun people pharmaceutical group co., Ltd]**. (1)Dried roots of Panax ginseng C. A. Mey., 20g; (2)Poria cocos (Schw) Wolf., 20g; (3)Dried rhizomes of Atractylodes macrocephala Koidz., 8g; (4)Aged fruit peel of Citrus reticulata Blanco., 20g., (5) Dried rhizomes of Rheum palmatum L., 12g; (6) Dried root peels of Lycium chinense Mill., 23g., (7) Dried roots of Glehnia littoralis Fr. Schmidtex Miq., 39g; (8) Dried rhizomes of Glycyrrhiza uralensis Fisch., 12g; (9)Artemisia annua L., 29g; (10) Dried roots of Ophiopogon japonicus (L.f) Ker-Gawl., 39g; (11) Dried twigs of Cinnamomum cassia Presl., 8g; (12) Dried rhizomes of Zingiber officinale Rose., 8g; (13) Dried roots of Aconitum carmichaelii Debx., 8g; (14)Dried fruits of Trichmanthes kiriloxvii Maxim., 29g; (15)Dried flower buds of Tussilago farfara L., 20g; (16) Dried rhizomes of Aster tataricus L. f., 20g; (17) Dried root peels of Morus alba L., 23g; (18) Dried roots of Astragalus membranaceus (Fisch.)Bge., 20g; (19)Dried fruits of Lycium barbarum Mill., 20g; (20) Dried root powder of Arisaema erubescens (Wall.) Schott with (ox bile or sheep bile or pig bile)., 8g; (21) Dried stomach lining of Callus gallus domesticus Brisson., 20g; (22) Dried turtle shell of Trionyx sinensis Wiegmann., 20g	Y-Prepared according to Chinese pharmacopoeia	Y - HPLC
Xiao and Li, 2016	**XFG, [Tian-sheng pharmaceutical group co., Ltd]**. (1)Dried roots of Panax ginseng C. A. Mey., 20g; (2)Poria cocos (Schw) Wolf., 20g; (3)Dried rhizomes of Atractylodes macrocephala Koidz., 8g; (4)Aged fruit peel of Citrus reticulata Blanco., 20g., (5) Dried rhizomes of Rheum palmatum L., 12g; (6) Dried root peels of Lycium chinense Mill., 23g., (7) Dried roots of Glehnia littoralis Fr. Schmidtex Miq., 39g; (8) Dried rhizomes of Glycyrrhiza uralensis Fisch., 12g; (9)Artemisia annua L., 29g; (10) Dried roots of Ophiopogon japonicus (L.f) Ker-Gawl., 39g; (11) Dried twigs of Cinnamomum cassia Presl., 8g; (12) Dried rhizomes of Zingiber officinale Rose., 8g; (13) Dried roots of Aconitum carmichaelii Debx., 8g; (14)Dried fruits of Trichmanthes kiriloxvii Maxim., 29g; (15)Dried flower buds of Tussilago farfara L., 20g; (16) Dried rhizomes of Aster tataricus L. f., 20g; (17) Dried root peels of Morus alba L., 23g; (18) Dried roots of Astragalus membranaceus (Fisch.)Bge., 20g; (19)Dried fruits of Lycium barbarum Mill., 20g; (20) Dried root powder of Arisaema erubescens (Wall.) Schott with (ox bile or sheep bile or pig bile)., 8g; (21) Dried stomach lining of Callus gallus domesticus Brisson., 20g; (22) Dried turtle shell of Trionyx sinensis Wiegmann., 20g	Y-Prepared according to Chinese pharmacopoeia	Y - HPLC
Yan, 2015	**XFG, [Tian-sheng pharmaceutical group co., Ltd or Chang-chun people pharmaceutical group co., Ltd]**. (1)Dried roots of Panax ginseng C. A. Mey., 20g; (2)Poria cocos (Schw) Wolf., 20g; (3)Dried rhizomes of Atractylodes macrocephala Koidz., 8g; (4)Aged fruit peel of Citrus reticulata Blanco., 20g., (5) Dried rhizomes of Rheum palmatum L., 12g; (6) Dried root peels of Lycium chinense Mill., 23g., (7) Dried roots of Glehnia littoralis Fr. Schmidtex Miq., 39g; (8) Dried rhizomes of Glycyrrhiza uralensis Fisch., 12g; (9)Artemisia annua L., 29g; (10) Dried roots of Ophiopogon japonicus (L.f) Ker-Gawl., 39g; (11) Dried twigs of Cinnamomum cassia Presl., 8g; (12) Dried rhizomes of Zingiber officinale Rose., 8g; (13) Dried roots of Aconitum carmichaelii Debx., 8g; (14)Dried fruits of Trichmanthes kiriloxvii Maxim., 29g; (15)Dried flower buds of Tussilago farfara L., 20g; (16) Dried rhizomes of Aster tataricus L. f., 20g; (17) Dried root peels of Morus alba L., 23g; (18) Dried roots of Astragalus membranaceus (Fisch.)Bge., 20g; (19)Dried fruits of Lycium barbarum Mill., 20g; (20) Dried root powder of Arisaema erubescens (Wall.) Schott with (ox bile or sheep bile or pig bile)., 8g; (21) Dried stomach lining of Callus gallus domesticus Brisson., 20g; (22) Dried turtle shell of Trionyx sinensis Wiegmann., 20g	Y-Prepared according to Chinese pharmacopoeia	Y - HPLC
Yan, 2017	**XFG, [Tian-sheng pharmaceutical group co., Ltd or Chang-chun people pharmaceutical group co., Ltd]**. (1)Dried roots of Panax ginseng C. A. Mey., 20g; (2)Poria cocos (Schw) Wolf., 20g; (3)Dried rhizomes of Atractylodes macrocephala Koidz., 8g; (4)Aged fruit peel of Citrus reticulata Blanco., 20g., (5) Dried rhizomes of Rheum palmatum L., 12g; (6) Dried root peels of Lycium chinense Mill., 23g., (7) Dried roots of Glehnia littoralis Fr. Schmidtex Miq., 39g; (8) Dried rhizomes of Glycyrrhiza uralensis Fisch., 12g; (9)Artemisia annua L., 29g; (10) Dried roots of Ophiopogon japonicus (L.f) Ker-Gawl., 39g; (11) Dried twigs of Cinnamomum cassia Presl., 8g; (12) Dried rhizomes of Zingiber officinale Rose., 8g; (13) Dried roots of Aconitum carmichaelii Debx., 8g; (14)Dried fruit of Trichmanthes kiriloxvii Maxim., 29g; (15)Dried flower buds of Tussilago farfara L., 20g; (16) Dried rhizomes of Aster tataricus L. f., 20g; (17) Dried root peels of Morus alba L., 23g; (18) Dried roots of Astragalus membranaceus (Fisch.)Bge., 20g; (19)Dried fruits of Lycium barbarum Mill., 20g; (20) Dried root powder of Arisaema erubescens (Wall.) Schott with (ox bile or sheep bile or pig bile)., 8g; (21) Dried stomach lining of Callus gallus domesticus Brisson., 20g; (22) Dried turtle shell of Trionyx sinensis Wiegmann., 20g	Y-Prepared according to Chinese pharmacopoeia	Y - HPLC
Zhang J. Q. et al., 2018	**XFG, [Chang-chun people pharmaceutical group co., Ltd]**. (1)Dried roots of Panax ginseng C. A. Mey., 20g; (2)Poria cocos (Schw) Wolf., 20g; (3)Dried rhizomes of Atractylodes macrocephala Koidz., 8g; (4)Aged fruit peel of Citrus reticulata Blanco., 20g., (5) Dried rhizomes of Rheum palmatum L., 12g; (6) Dried root peels of Lycium chinense Mill., 23g., (7) Dried roots of Glehnia littoralis Fr. Schmidtex Miq., 39g; (8) Dried rhizomes of Glycyrrhiza uralensis Fisch., 12g; (9)Artemisia annua L., 29g; (10) Dried roots of Ophiopogon japonicus (L.f) Ker-Gawl., 39g; (11) Dried twigs of Cinnamomum cassia Presl., 8g; (12) Dried rhizomes of Zingiber officinale Rose., 8g; (13) Dried roots of Aconitum carmichaelii Debx., 8g; (14)Dried fruits of Trichmanthes kiriloxvii Maxim., 29g; (15)Dried flower buds of Tussilago farfara L., 20g; (16) Dried rhizomes of Aster tataricus L. f., 20g; (17) Dried root peels of Morus alba L., 23g; (18) Dried roots of Astragalus membranaceus (Fisch.)Bge., 20g; (19)Dried fruits of Lycium barbarum Mill., 20g; (20) Dried root powder of Arisaema erubescens (Wall.) Schott with (ox bile or sheep bile or pig bile)., 8g; (21) Dried stomach lining of Callus gallus domesticus Brisson., 20g; (22) Dried turtle shell of Trionyx sinensis Wiegmann., 20g	Y-Prepared according to Chinese pharmacopoeia	Y - HPLC
Zhang and Guo, 2018	**XFG, [Tian-sheng pharmaceutical group co., Ltd]**. (1)Dried roots of Panax ginseng C. A. Mey., 20g; (2)Poria cocos (Schw) Wolf., 20g; (3)Dried rhizomes of Atractylodes macrocephala Koidz., 8g; (4)Aged fruit peel of Citrus reticulata Blanco., 20g., (5) Dried rhizomes of Rheum palmatum L., 12g; (6) Dried root peels of Lycium chinense Mill., 23g., (7) Dried roots of Glehnia littoralis Fr. Schmidtex Miq., 39g; (8) Dried rhizomes of Glycyrrhiza uralensis Fisch., 12g; (9)Artemisia annua L., 29g; (10) Dried roots of Ophiopogon japonicus (L.f) Ker-Gawl., 39g; (11) Dried twigs of Cinnamomum cassia Presl., 8g; (12) Dried rhizomes of Zingiber officinale Rose., 8g; (13) Dried roots of Aconitum carmichaelii Debx., 8g; (14)Dried fruits of Trichmanthes kiriloxvii Maxim., 29g; (15)Dried flower buds of Tussilago farfara L., 20g; (16) Dried rhizomes of Aster tataricus L. f., 20g; (17) Dried root peels of Morus alba L., 23g; (18) Dried roots of Astragalus membranaceus (Fisch.)Bge., 20g; (19)Dried fruits of Lycium barbarum Mill., 20g; (20) Dried root powder of Arisaema erubescens (Wall.) Schott with (ox bile or sheep bile or pig bile)., 8g; (21) Dried stomach lining of Callus gallus domesticus Brisson., 20g; (22) Dried turtle shell of Trionyx sinensis Wiegmann., 20g	Y-Prepared according to Chinese pharmacopoeia	Y - HPLC
He and Yang, 2018	**XFG, [Chang-chun people pharmaceutical group co., Ltd]**. (1)Dried roots of Panax ginseng C. A. Mey., 20g; (2)Poria cocos (Schw) Wolf., 20g; (3)Dried rhizomes of Atractylodes macrocephala Koidz., 8g; (4)Aged fruit peel of Citrus reticulata Blanco., 20g., (5) Dried rhizomes of Rheum palmatum L., 12g; (6) Dried root peels of Lycium chinense Mill., 23g., (7) Dried roots of Glehnia littoralis Fr. Schmidtex Miq., 39g; (8) Dried rhizomes of Glycyrrhiza uralensis Fisch., 12g; (9)Artemisia annua L., 29g; (10) Dried roots of Ophiopogon japonicus (L.f) Ker-Gawl., 39g; (11) Dried twigs of Cinnamomum cassia Presl., 8g; (12) Dried rhizomes of Zingiber officinale Rose., 8g; (13) Dried roots of Aconitum carmichaelii Debx., 8g; (14)Dried fruits of Trichmanthes kiriloxvii Maxim., 29g; (15)Dried flower buds of Tussilago farfara L., 20g; (16) Dried rhizomes of Aster tataricus L. f., 20g; (17) Dried root peels of Morus alba L., 23g; (18) Dried roots of Astragalus membranaceus (Fisch.)Bge., 20g; (19)Dried fruits of Lycium barbarum Mill., 20g; (20) Dried root powder of Arisaema erubescens (Wall.) Schott with (ox bile or sheep bile or pig bile)., 8g; (21) Dried stomach lining of Callus gallus domesticus Brisson., 20g; (22) Dried turtle shell of Trionyx sinensis Wiegmann., 20g	Y-Prepared according to Chinese pharmacopoeia	Y - HPLC
Hu et al., 2018	**XFG, [Tian-sheng pharmaceutical group co., Ltd]**. (1)Dried roots of Panax ginseng C. A. Mey., 20g; (2)Poria cocos (Schw) Wolf., 20g; (3)Dried rhizomes of Atractylodes macrocephala Koidz., 8g; (4)Aged fruit peel of Citrus reticulata Blanco., 20g., (5) Dried rhizomes of Rheum palmatum L., 12g; (6) Dried root peels of Lycium chinense Mill., 23g., (7) Dried roots of Glehnia littoralis Fr. Schmidtex Miq., 39g; (8) Dried rhizomes of Glycyrrhiza uralensis Fisch., 12g; (9)Artemisia annua L., 29g; (10) Dried roots of Ophiopogon japonicus (L.f) Ker-Gawl., 39g; (11) Dried twigs of Cinnamomum cassia Presl., 8g; (12) Dried rhizomes of Zingiber officinale Rose., 8g; (13) Dried roots of Aconitum carmichaelii Debx., 8g; (14)Dried fruits of Trichmanthes kiriloxvii Maxim., 29g; (15)Dried flower buds of Tussilago farfara L., 20g; (16) Dried rhizomes of Aster tataricus L. f., 20g; (17) Dried root peels of Morus alba L., 23g; (18) Dried roots of Astragalus membranaceus (Fisch.)Bge., 20g; (19)Dried fruits of Lycium barbarum Mill., 20g; (20) Dried root powder of Arisaema erubescens (Wall.) Schott with (ox bile or sheep bile or pig bile)., 8g; (21) Dried stomach lining of Callus gallus domesticus Brisson., 20g; (22) Dried turtle shell of Trionyx sinensis Wiegmann., 20g	Y-Prepared according to Chinese pharmacopoeia	Y - HPLC
Li, 2015	**XFG, [Tian-sheng pharmaceutical group co., Ltd]**. (1)Dried roots of Panax ginseng C. A. Mey., 20g; (2)Poria cocos (Schw) Wolf., 20g; (3)Dried rhizomes of Atractylodes macrocephala Koidz., 8g; (4)Aged fruit peel of Citrus reticulata Blanco., 20g., (5) Dried rhizomes of Rheum palmatum L., 12g; (6) Dried root peels of Lycium chinense Mill., 23g., (7) Dried roots of Glehnia littoralis Fr. Schmidtex Miq., 39g; (8) Dried rhizomes of Glycyrrhiza uralensis Fisch., 12g; (9)Artemisia annua L., 29g; (10) Dried roots of Ophiopogon japonicus (L.f) Ker-Gawl., 39g; (11) Dried twigs of Cinnamomum cassia Presl., 8g; (12) Dried rhizomes of Zingiber officinale Rose., 8g; (13) Dried roots of Aconitum carmichaelii Debx., 8g; (14)Dried fruits of Trichmanthes kiriloxvii Maxim., 29g; (15)Dried flower buds of Tussilago farfara L., 20g; (16) Dried rhizomes of Aster tataricus L. f., 20g; (17) Dried root peels of Morus alba L., 23g; (18) Dried roots of Astragalus membranaceus (Fisch.)Bge., 20g; (19)Dried fruits of Lycium barbarum Mill., 20g; (20) Dried root powder of Arisaema erubescens (Wall.) Schott with (ox bile or sheep bile or pig bile)., 8g; (21) Dried stomach lining of Callus gallus domesticus Brisson., 20g; (22) Dried turtle shell of Trionyx sinensis Wiegmann., 20g	Y-Prepared according to Chinese pharmacopoeia	Y - HPLC
Liao and Lou, 2018	**XFG, [Tian-sheng pharmaceutical group co., Ltd]**. (1)Dried roots of Panax ginseng C. A. Mey., 20g; (2)Poria cocos (Schw) Wolf., 20g; (3)Dried rhizomes of Atractylodes macrocephala Koidz., 8g; (4)Aged fruit peel of Citrus reticulata Blanco., 20g., (5) Dried rhizomes of Rheum palmatum L., 12g; (6) Dried root peels of Lycium chinense Mill., 23g., (7) Dried roots of Glehnia littoralis Fr. Schmidtex Miq., 39g; (8) Dried rhizomes of Glycyrrhiza uralensis Fisch., 12g; (9)Artemisia annua L., 29g; (10) Dried roots of Ophiopogon japonicus (L.f) Ker-Gawl., 39g; (11) Dried twigs of Cinnamomum cassia Presl., 8g; (12) Dried rhizomes of Zingiber officinale Rose., 8g; (13) Dried roots of Aconitum carmichaelii Debx., 8g; (14)Dried fruits of Trichmanthes kiriloxvii Maxim., 29g; (15)Dried flower buds of Tussilago farfara L., 20g; (16) Dried rhizomes of Aster tataricus L. f., 20g; (17) Dried root peels of Morus alba L., 23g; (18) Dried roots of Astragalus membranaceus (Fisch.)Bge., 20g; (19)Dried fruits of Lycium barbarum Mill., 20g; (20) Dried root powder of Arisaema erubescens (Wall.) Schott with (ox bile or sheep bile or pig bile)., 8g; (21) Dried stomach lining of Callus gallus domesticus Brisson., 20g; (22) Dried turtle shell of Trionyx sinensis Wiegmann., 20g	Y-Prepared according to Chinese pharmacopoeia	Y - HPLC
Xu et al., 2015	**XFG, [Chang-chun people pharmaceutical group co., Ltd]**. (1)Dried roots of Panax ginseng C. A. Mey., 20g; (2)Poria cocos (Schw) Wolf., 20g; (3)Dried rhizomes of Atractylodes macrocephala Koidz., 8g; (4)Aged fruit peel of Citrus reticulata Blanco., 20g., (5) Dried rhizomes of Rheum palmatum L., 12g; (6) Dried root peels of Lycium chinense Mill., 23g., (7) Dried roots of Glehnia littoralis Fr. Schmidtex Miq., 39g; (8) Dried rhizomes of Glycyrrhiza uralensis Fisch., 12g; (9)Artemisia annua L., 29g; (10) Dried roots of Ophiopogon japonicus (L.f) Ker-Gawl., 39g; (11) Dried twigs of Cinnamomum cassia Presl., 8g; (12) Dried rhizomes of Zingiber officinale Rose., 8g; (13) Dried roots of Aconitum carmichaelii Debx., 8g; (14)Dried fruits of Trichmanthes kiriloxvii Maxim., 29g; (15)Dried flower buds of Tussilago farfara L., 20g; (16) Dried rhizomes of Aster tataricus L. f., 20g; (17) Dried root peels of Morus alba L., 23g; (18) Dried roots of Astragalus membranaceus (Fisch.)Bge., 20g; (19)Dried fruits of Lycium barbarum Mill., 20g; (20) Dried root powder of Arisaema erubescens (Wall.) Schott with (ox bile or sheep bile or pig bile)., 8g; (21) Dried stomach lining of Callus gallus domesticus Brisson., 20g; (22) Dried turtle shell of Trionyx sinensis Wiegmann., 20g	Y-Prepared according to Chinese pharmacopoeia	Y - HPLC
Ye and Zhen, 2016	**XFG, [Tian-sheng pharmaceutical group co., Ltd]**. (1)Dried roots of Panax ginseng C. A. Mey., 20g; (2)Poria cocos (Schw) Wolf., 20g; (3)Dried rhizomes of Atractylodes macrocephala Koidz., 8g; (4)Aged fruit peel of Citrus reticulata Blanco., 20g., (5) Dried rhizomes of Rheum palmatum L., 12g; (6) Dried root peels of Lycium chinense Mill., 23g., (7) Dried roots of Glehnia littoralis Fr. Schmidtex Miq., 39g; (8) Dried rhizomes of Glycyrrhiza uralensis Fisch., 12g; (9)Artemisia annua L., 29g; (10) Dried roots of Ophiopogon japonicus (L.f) Ker-Gawl., 39g; (11) Dried twigs of Cinnamomum cassia Presl., 8g; (12) Dried rhizomes of Zingiber officinale Rose., 8g; (13) Dried roots of Aconitum carmichaelii Debx., 8g; (14)Dried fruits of Trichmanthes kiriloxvii Maxim., 29g; (15)Dried flower buds of Tussilago farfara L., 20g; (16) Dried rhizomes of Aster tataricus L. f., 20g; (17) Dried root peels of Morus alba L., 23g; (18) Dried roots of Astragalus membranaceus (Fisch.)Bge., 20g; (19)Dried fruits of Lycium barbarum Mill., 20g; (20) Dried root powder of Arisaema erubescens (Wall.) Schott with (ox bile or sheep bile or pig bile)., 8g; (21) Dried stomach lining of Callus gallus domesticus Brisson., 20g; (22) Dried turtle shell of Trionyx sinensis Wiegmann., 20g	Y-Prepared according to Chinese pharmacopoeia	Y - HPLC
Zhao and Chen, 2017	**XFG, [Tian-sheng pharmaceutical group co., Ltd]**. (1)Dried roots of Panax ginseng C. A. Mey., 20g; (2)Poria cocos (Schw) Wolf., 20g; (3)Dried rhizomes of Atractylodes macrocephala Koidz., 8g; (4)Aged fruit peel of Citrus reticulata Blanco., 20g., (5) Dried rhizomes of Rheum palmatum L., 12g; (6) Dried root peels of Lycium chinense Mill., 23g., (7) Dried roots of Glehnia littoralis Fr. Schmidtex Miq., 39g; (8) Dried rhizomes of Glycyrrhiza uralensis Fisch., 12g; (9)Artemisia annua L., 29g; (10) Dried roots of Ophiopogon japonicus (L.f) Ker-Gawl., 39g; (11) Dried twigs of Cinnamomum cassia Presl., 8g; (12) Dried rhizomes of Zingiber officinale Rose., 8g; (13) Dried roots of Aconitum carmichaelii Debx., 8g; (14)Dried fruits of Trichmanthes kiriloxvii Maxim., 29g; (15)Dried flower buds of Tussilago farfara L., 20g; (16) Dried rhizomes of Aster tataricus L. f., 20g; (17) Dried root peels of Morus alba L., 23g; (18) Dried roots of Astragalus membranaceus (Fisch.)Bge., 20g; (19)Dried fruits of Lycium barbarum Mill., 20g; (20) Dried root powder of Arisaema erubescens (Wall.) Schott with (ox bile or sheep bile or pig bile)., 8g; (21) Dried stomach lining of Callus gallus domesticus Brisson., 20g; (22) Dried turtle shell of Trionyx sinensis Wiegmann., 20g	Y-Prepared according to Chinese pharmacopoeia	Y - HPLC

### Primary Chemical Compositions

HPLC analysis was performed to determine the primary chemical compositions of XFG. We determined a total of 17 chemical compounds (hesperidin, nobiletin, tangeretin, aoleemodin, emodin, rhein, chrysophanol, physcion, ginsenoside Rg1, ginsenoside Re, ginsenoside Rb1, atractylenolide I, atractylenolide III, shionone, chlorogenic acid, scopoletin, and tussilagone). After a comparison with the respective reference samples, fourteen potential active compounds were detected, as shown in **Table 5**.

**Table 5:** Primary chemical compositions of XFG.

Compositions	Chemical structure	Molecular Formula	PubMed CID	CAS no	HPLC
Hesperidin	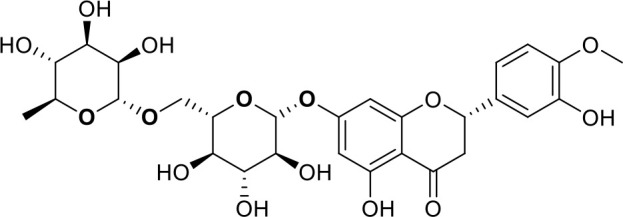	C_28_H_34_O_15_	10621	520-26-3	Agilent 1290
Nobiletin	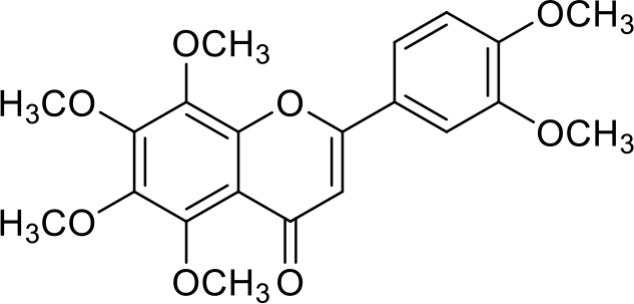	C_21_H_22_O_8_	72344	478-01-3	Agilent 1290
Tangeretin	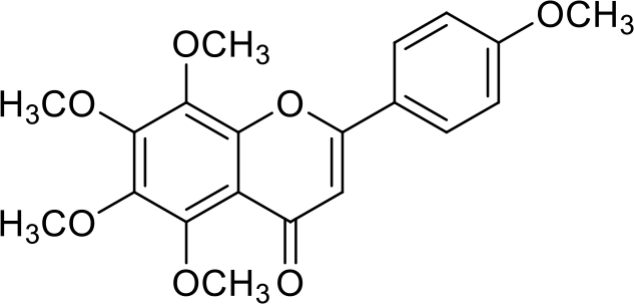	C_20_H_20_O_7_	68077	481-53-8	Agilent 1290
Aoleemodin	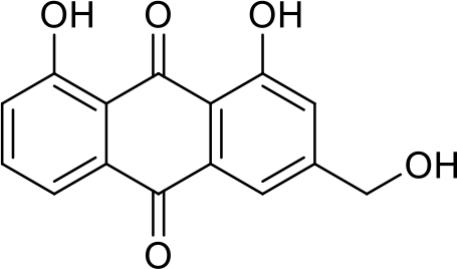	C_15_H_10_O_5_	10207	481-72-1	Agilent 1290
Emodin	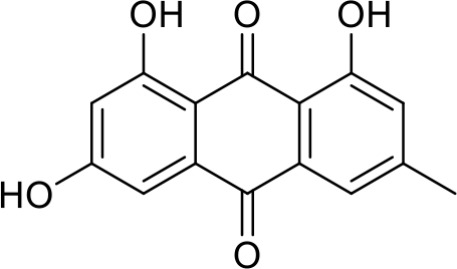	C_15_H_10_O_5_	3220	518-82-1	Agilent 1290
Chrysophanol	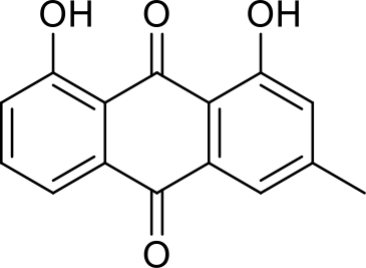	C_15_H_10_O_4_	10208	481-74-3	Agilent 1290
Physcion	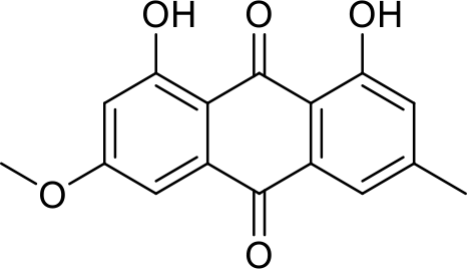	C_16_H_12_O_5_	10639	521-61-9	Agilent 1290
Ginsenoside Re	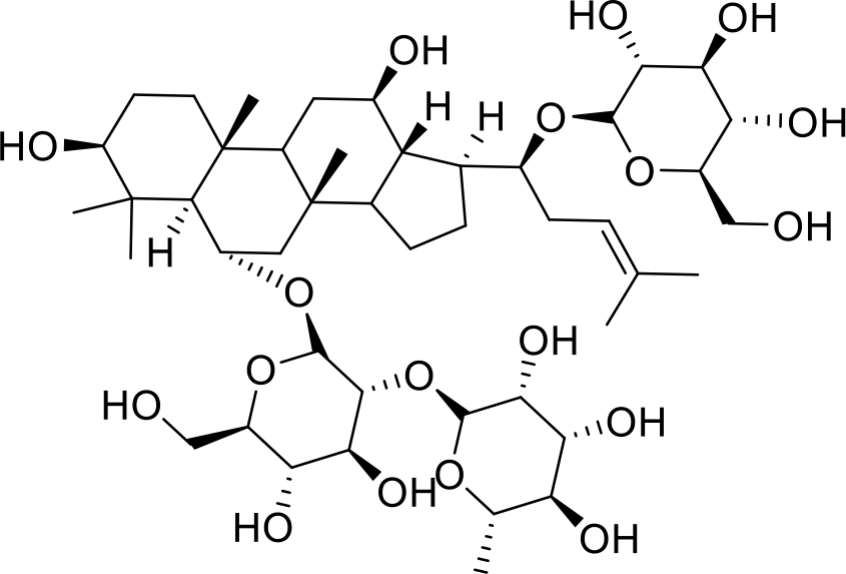	C_48_H_82_O_18_	441921	51542-56-4	Agilent 1290
Ginsenoside Rb1	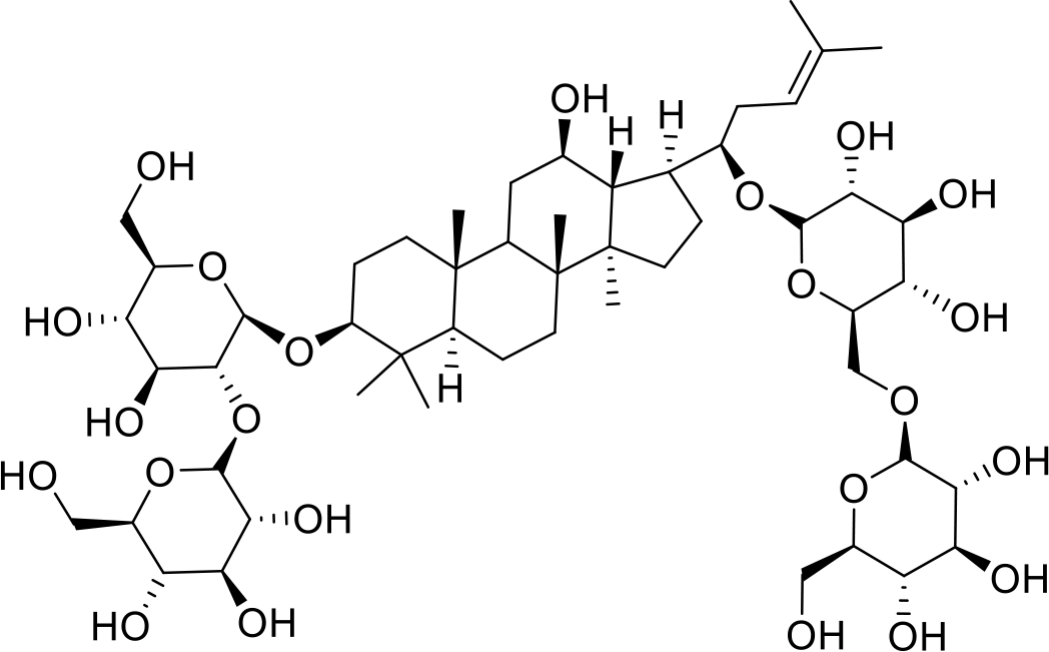	C_54_H_92_O_23_	9898279	41753-43-9	Agilent 1290
Atractylenolide I	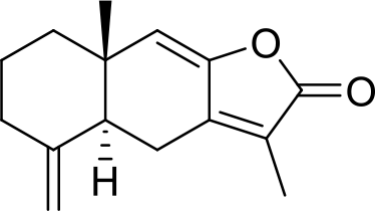	C15H18O2	5321018	73069-13-3	Agilent 1260
Shionone	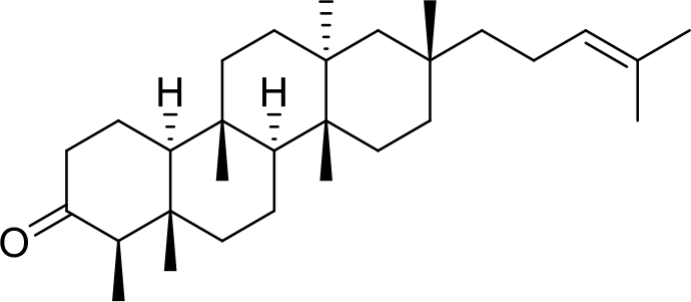	C_30_H_50_O	12315507	10376-48-4	Agilent 1260
Tussilagone	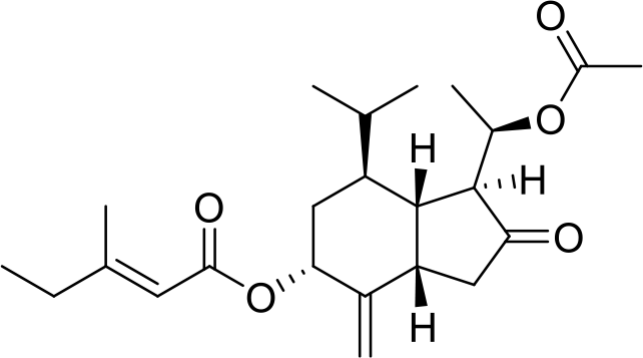	C_23_H_34_O_5_	71307581	104012-37-5	Agilent 1290
Chlorogenic acid	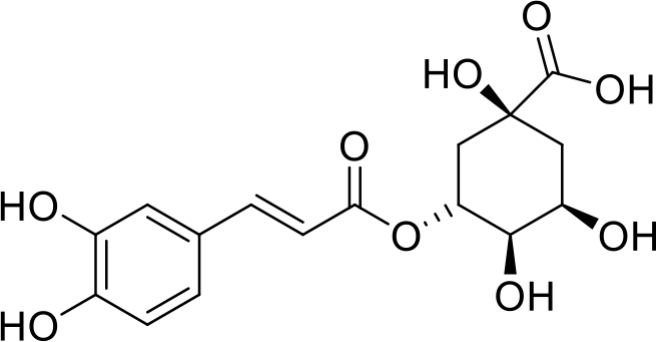	C_16_H_18_O_9_	1794427	327-97-9	Agilent 1260
Scopoletin	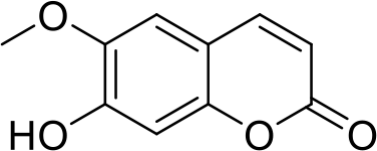	C_10_H_8_O_4_	5280460	92-61-5	Agilent 1260

### Implications for Further Study

This review has evaluated the curative effects of XFG, but most of the included studies used CER, RTC, RTR, and RTF as the primary outcomes. A few studies reported on the immune cells (CD4, CD8, and WBC) and cytokine levels (TNF-α, INF-γ, IL-4, IL-6, IL-8, IL-10, and CRP, etc.), (Li, 2015; Du, 2016; Ma and Lu, 2017; Zhao and Chen, 2017; Hu et al., 2018; Zhang and Guo, 2018; Zhang J. Q. et al., 2018). This indicates that the therapeutic mechanisms of XFG are associated with immune enhancement and inflammation reduction. However, this explanation is far from conclusive, because these cellular and molecular parameters were only reported in three studies. The therapeutic effects of XFG are multiple-target, multi-level, and holistic, and therefore, at the cellular and molecular levels, the mechanisms of action of XFG warrant further investigation. Although the evidence analyzed in this study indicated that XFG is an effective and safe adjuvant therapy in treating ALRI, we found significant heterogeneity and publication bias. Therefore, high-quality, multicenter, double-blind RCTs are needed to provide reliable evidence that will guide the rational use of XFG in clinical settings.

### Limitations

Several limitations should be highlighted in our meta-analysis. First, we searched only the primary English and Chinese databases. Therefore, some studies that meet our inclusion criteria in other languages or databases may have been excluded. All the included trials declared randomizations, but only eleven studies described a specific randomization method. Blinded assessments were not detailed in all the included documents, which may have exerted a potential impact on the objectivity of the ALRI outcomes. Second, the inclusion criteria of these studies had small sample sizes with low-quality designs, which may have led them to overvalue the benefits of XFG. Additionally, there may be a degree of selective reporting bias because the majority of studies were not officially registered. Third, only a few eligible studies reported the HS, RTIL, cellular, and cytokine outcomes. Fourth, six papers did not mention any information on adverse reactions. Thus, insights as to the safety of using XFG to treat ALRI are limited. Fifth, although the RTC and RTR were widely used to evaluate the curative effect of XFG for ALRI in hospitalized patients, this was still somewhat subjective. Although these limitations may undermine the quality of the evidence, the trials are nevertheless comparable, and the documents were selected using strict inclusion criteria. Since the patients of the selected studies were primarily from China, the conclusion of this meta-analysis might not apply to other ethnic groups. Therefore, large sample trials of a higher-quality, with well-designed considerations of different ethnic groups should be conducted in the future to provide more reliable evidence regarding the efficacy and safety of using XFG to treat ALRI.

## Conclusion

The findings of this meta-analysis support the use of XFG in the treatment of ALRI. However, the results should be treated with caution due to the significant heterogeneity and publication bias of the studies examined. Therefore, further well-designed and high-quality RCTs are needed to interrogate the efficacy and safety of XFG.

## Data Availability Statement

The raw data supporting the conclusions of this article will be made available by the authors, without undue reservation, to any qualified researcher.

## Author Contributions

QY and LL conceived this review and completed the manuscript. DL performed the literature searches electronically and manually. DY and LC performed the study selection and data extraction and assessed the risk of bias. Y-pL and H-pC critically revised the paper. All authors contributed to the article and approved the submitted version.

## Funding

This study was funded by the National Natural Science Foundation of China (No.: 81973436) and Research Premotion Project of Chengdu University of TCM (No: CXTD2018011).

## Conflict of Interest

The authors declare that the research was conducted in the absence of any commercial or financial relationships that could be construed as a potential conflict of interest.
