# Supplementary material for: Meta-Analysis on the Chinese Herbal Formula Xiaoer-Feike Granules as a Complementary Therapy for Children With Acute Lower Respiratory Infections
Source: Front Pharmacol. 2020 Oct 22;11:496348. doi: 10.3389/fphar.2020.496348 (PMC7642815; doi:10.3389/fphar.2020.496348)
Supplement: Supplementary file 1 [file DataSheet_1.pdf]

Meta-analysis on Chinese herbal formula *Xiaoer-Feike granule* as complementary therapy for children with acute lower respiratory infection

Methods. XFG was extracted by methanol and ultrasounic for 1h (220V, 500W, 50kHz) (Shanghai Gutel ultrasounic equipment co., LTD). Reference substances were dissolved in methanol. The compositions were detected by the UV detector using an Agilent 1260 and Agilent 1290 Series HPLC system (Agilent Technologies Inc., USA). The compounds were separated on an Agilent ZORBAX SB C18 column (4.6 mm ×250 mm, 5μm).

Chemicals. High purity water, HPLC-grade acetonitrile and methyl alcohol (TEDIA, Ohio, America) were used after filtration through a 0.2 μm membrane filter. HPLC-grade phosphoric acid (Shanghai Aladdin biochemical technology co., LTD, Shanghai, China).

TABLE 1 | the source and batch number of reference substances

Reference substance	Source	Batch number
Chrysophanol	Sichuan Weikeqi Biological Technology Co., Ltd, Chengdu, China	WKQ19012503
Atractylenolide I	Sichuan Weikeqi Biological Technology Co., Ltd, Chengdu, China	WKQ17062303
AtractylenolideIII	Sichuan Weikeqi Biological Technology Co., Ltd, Chengdu, China	WKQ16092604
Ginsenoside Re	Sichuan Weikeqi Biological Technology Co., Ltd, Chengdu, China	WKQ18121407
Ginsenoside Rb1	Sichuan Weikeqi Biological Technology Co., Ltd, Chengdu, China	WKQ19012107
Rhein	Sichuan Weikeqi Biological Technology Co., Ltd, Chengdu, China	WKQ19030402

Ginsenoside Rg1	Sichuan Weikeqi Biological Technology Co., Ltd, Chengdu, China	WKQ19040208
Shionone	Sichuan Weikeqi Biological Technology Co., Ltd, Chengdu, China	WKQ19042901
Tussilagone	Sichuan Weikeqi Biological Technology Co., Ltd, Chengdu, China	WKQ19042510
Hesperidin	Chengdu institute of biology, Chinese academy of sciences, Chengdu, China	MUST-18042205
Nobiletin	Chengdu institute of biology, Chinese academy of sciences, Chengdu, China	MUST-18032502
Tangeretin	Chengdu herbpurify biotechnology co., LTD, Chengdu, China	J-022-150730
Scopoletin	Chengdu herbpurify biotechnology co., LTD, Chengdu, China	—
Emodin	National Institutes for Food and Drug Control, Beijing, China	CVX4-E9JJ
Aloe-emodin	National Institutes for Food and Drug Control, Beijing, China	IQLS-EFVV
Physcion	National Institutes for Food and Drug Control, Beijing, China	110758-200610
Chlorogenic acid	Chengdu Chroma-biotechnology Co., Ltd, Chengdu, China	CHB170713

HPLC chromatograms

Hesperidin: Solvent system, acetonitrile and water with 0.1% phosphoric acid with gradient elution program: 0-10 min, 20-30% acetonitrile; 10-20 min, 30-50 % acetonitrile, 20-25 min, 50-52% acetonitrile, 25-30 min, 52% acetonitrile, 30-35 min, 52-95 % acetonitrile, 35-37 min, 95% acetonitrile; flow rate 0.7ml/min, UV detection wavelength 283nm.

A

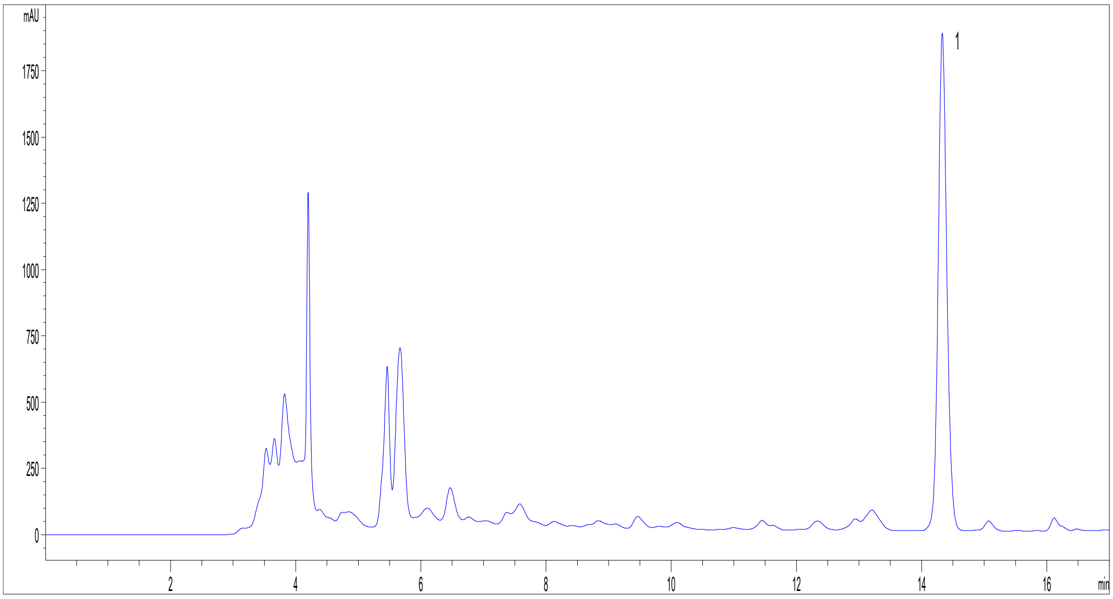

B

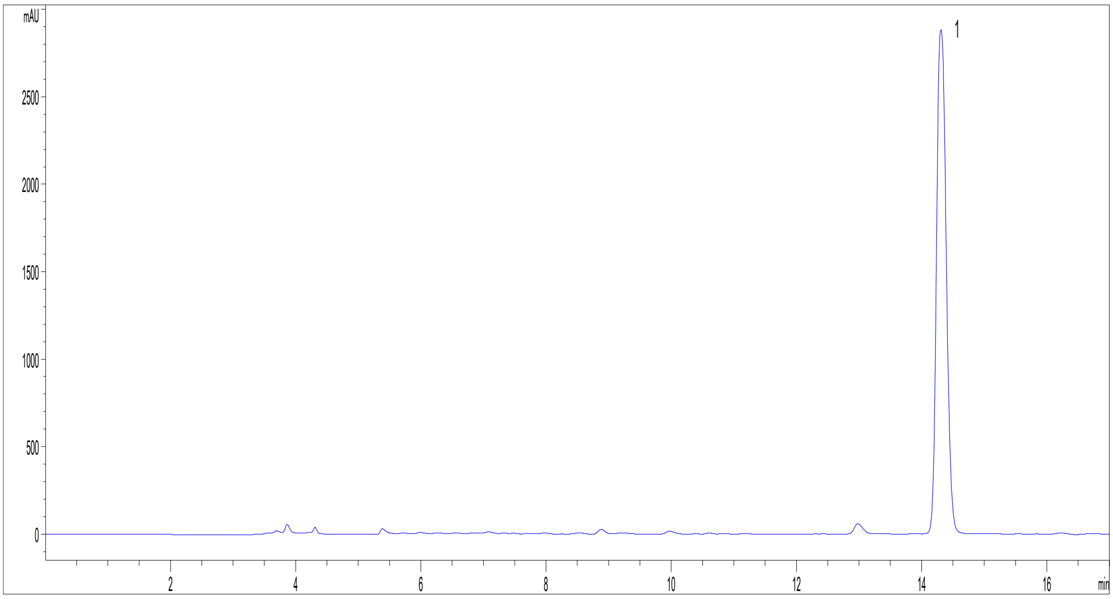

FIGRURE 1 | HPLC chromatograms of A: XFG methanol extract and B: Hesperidin reference

Nobiletin and Tangeretin: Solvent system, acetonitrile and water with 0.1% phosphoric acid with gradient elution program: 0-10 min, 20-30% acetonitrile; 10-20 min, 30-50 % acetonitrile, 20-25 min, 50-52% acetonitrile, 25-30 min, 52% acetonitrile, 30-35 min, 52-95 % acetonitrile, 35-37 min, 95% acetonitrile; flow rate 0.7ml/min, UV detection

wavelength 335nm.

A

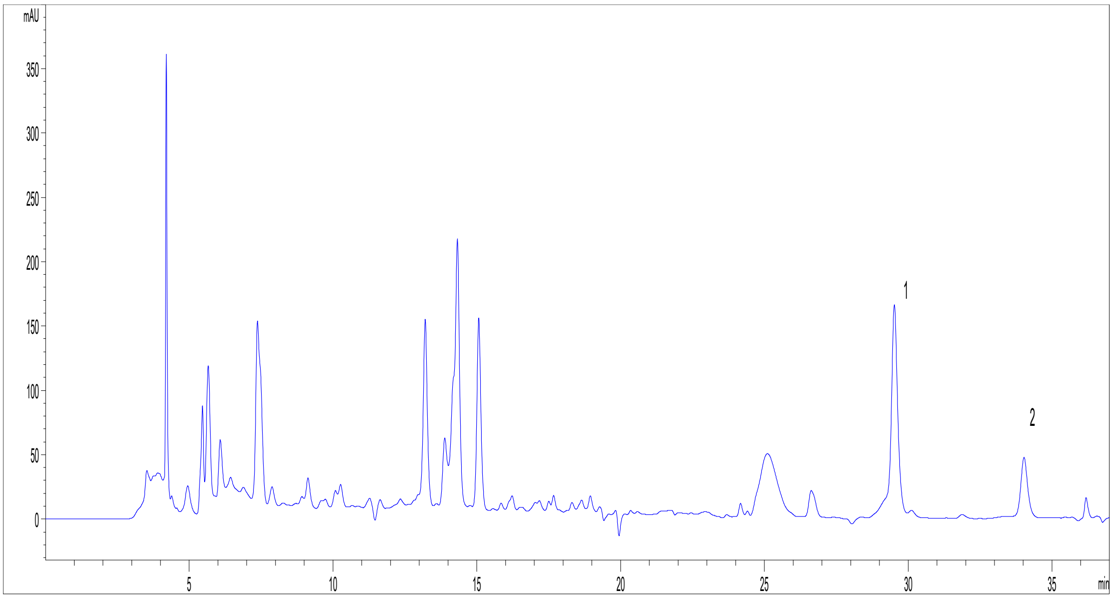

B

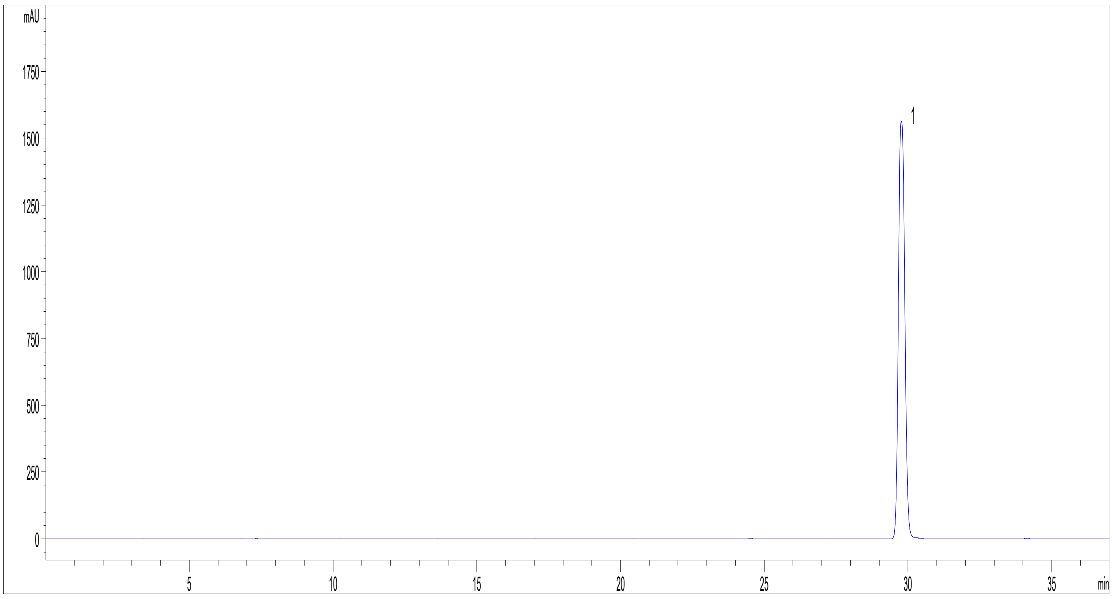

C

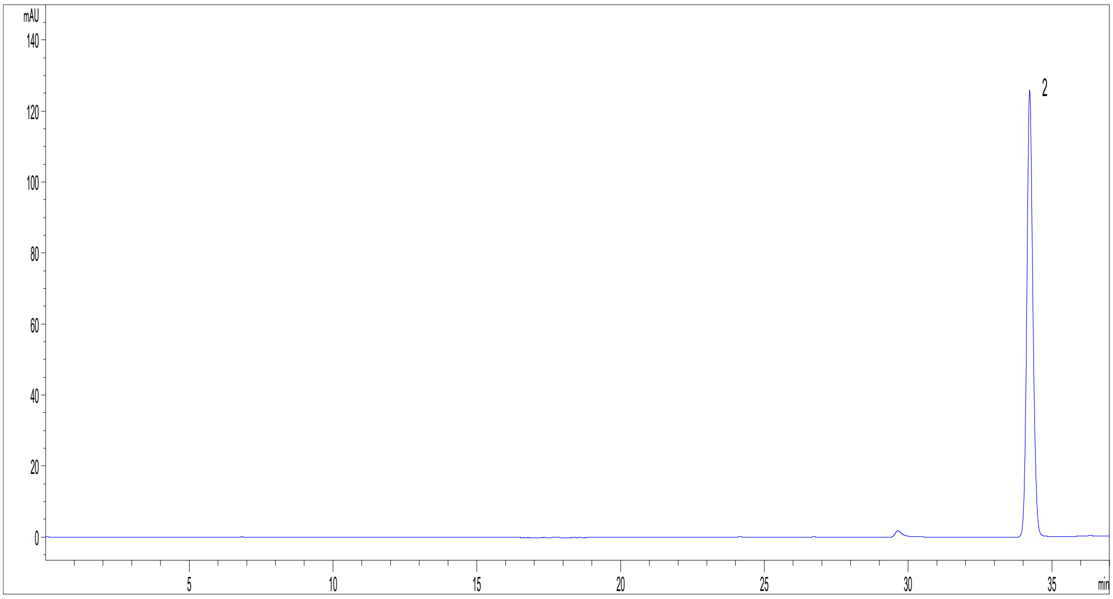

FIGRURE 2 | HPLC chromatograms of A: XFG methanol extract, B: Nobiletin reference and C: Tangeretin reference

Aoleemodin, rhein, emodin, chrysophanol and physcion: Solvent system, 78% methyl alcohol and 22% water with 0.1% phosphoric acid, flow rate 1.0 ml/min, UV detection wavelength 254 nm

A

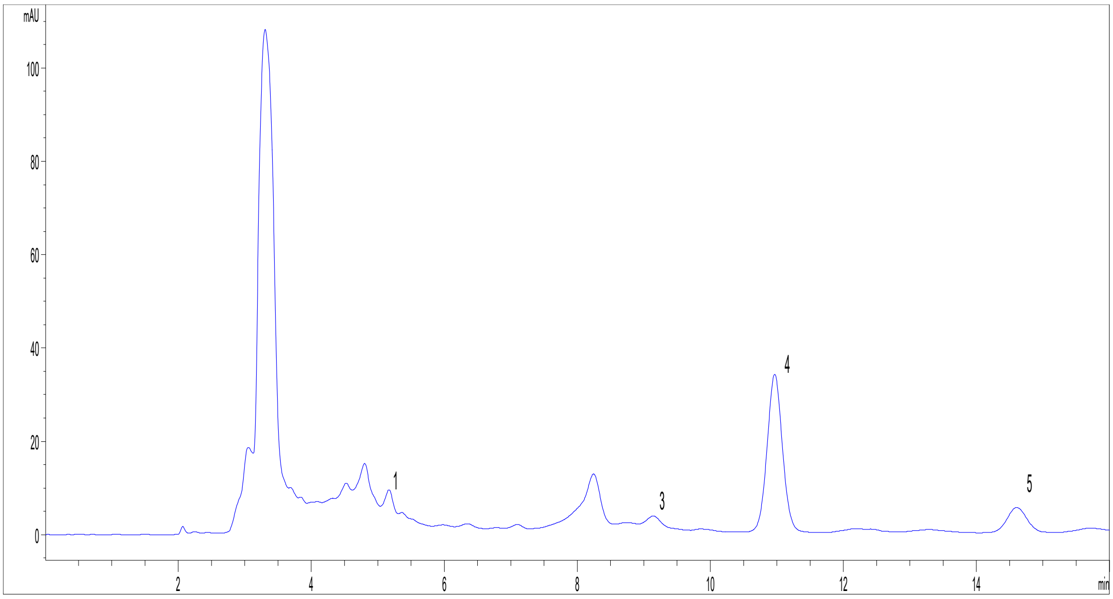

B

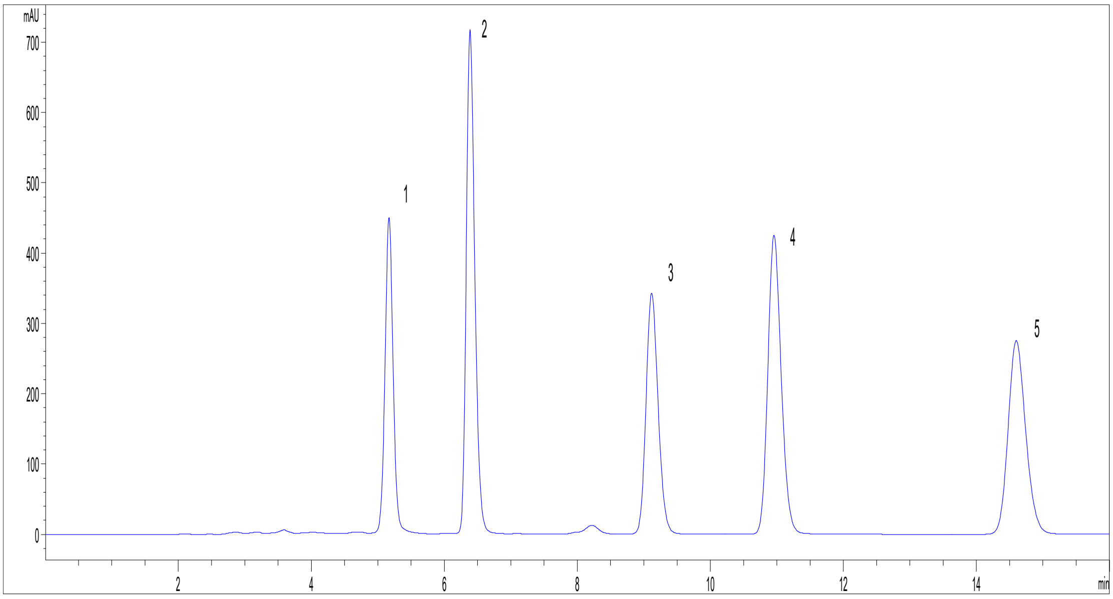

**FIGRURE 3 | ** HPLC chromatograms of A: XFG methanol extract, B1: Aoleemodin reference, B2: rhein reference, B3: emodin reference, B4: chrysophanol reference and B5: physcion reference

Ginsenoside Rg1, Ginsenoside Re and Ginsenoside Rb1: Solvent system, acetonitrile and water with gradient elution program: 0-35 min, 19% acetonitrile; 35-55 min, 19-29% acetonitrile, 55-70 min, 29% acetonitrile, and 70-100 min, 29-40% acetonitrile, flow rate 1.0 ml/min, UV detection wavelength 203 nm

A

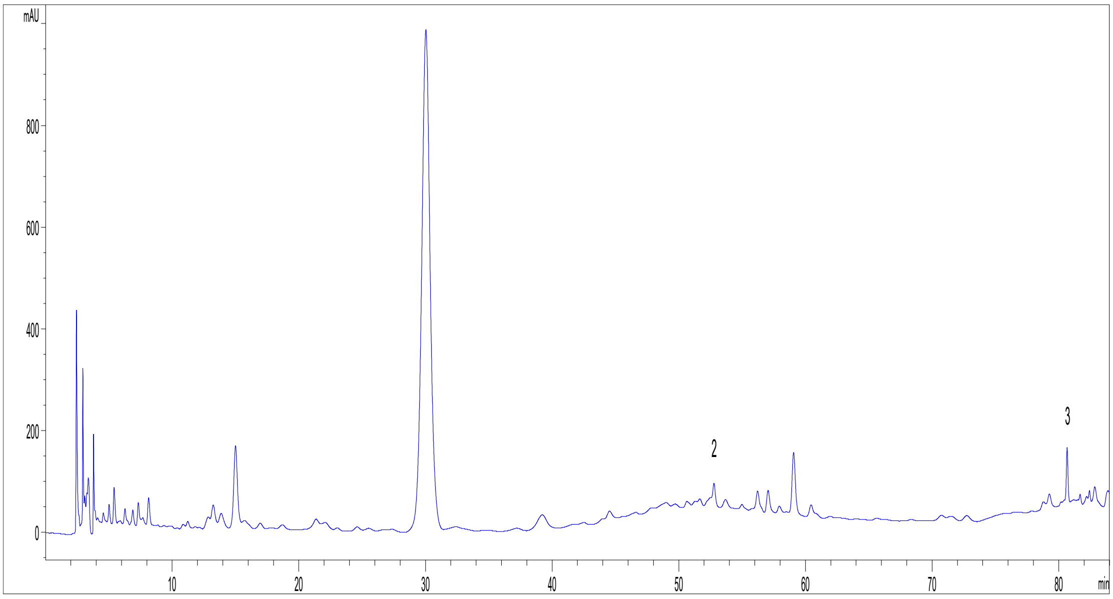

B

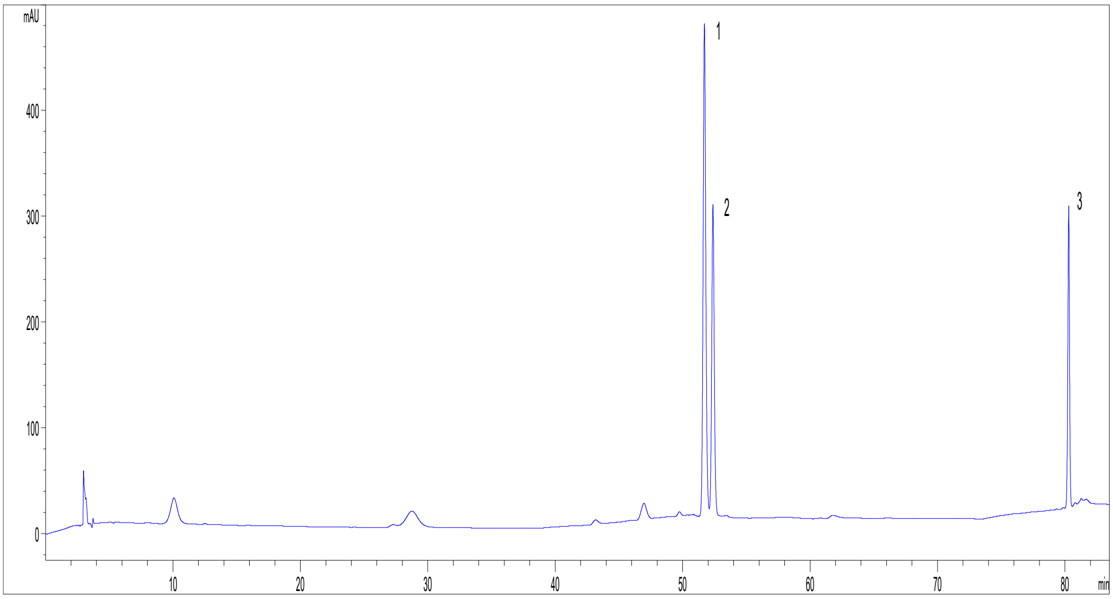

FIGRURE 4 | HPLC chromatograms of A: XFG methanol extract, B1: Ginsenoside Rg1 reference, B2:Ginsenoside Re reference and B3:Ginsenoside Rb1 reference

Tussilagone: Solvent system, 85% methyl alcohol and 15% water, flow rate 1.0 ml/min, UV detection wavelength 220 nm

A

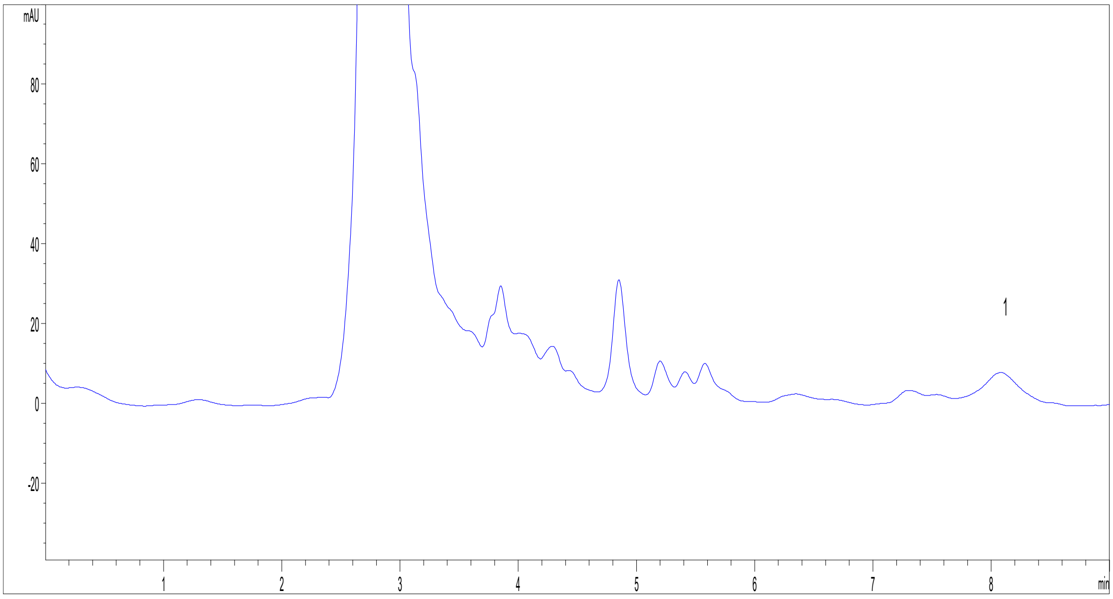

B

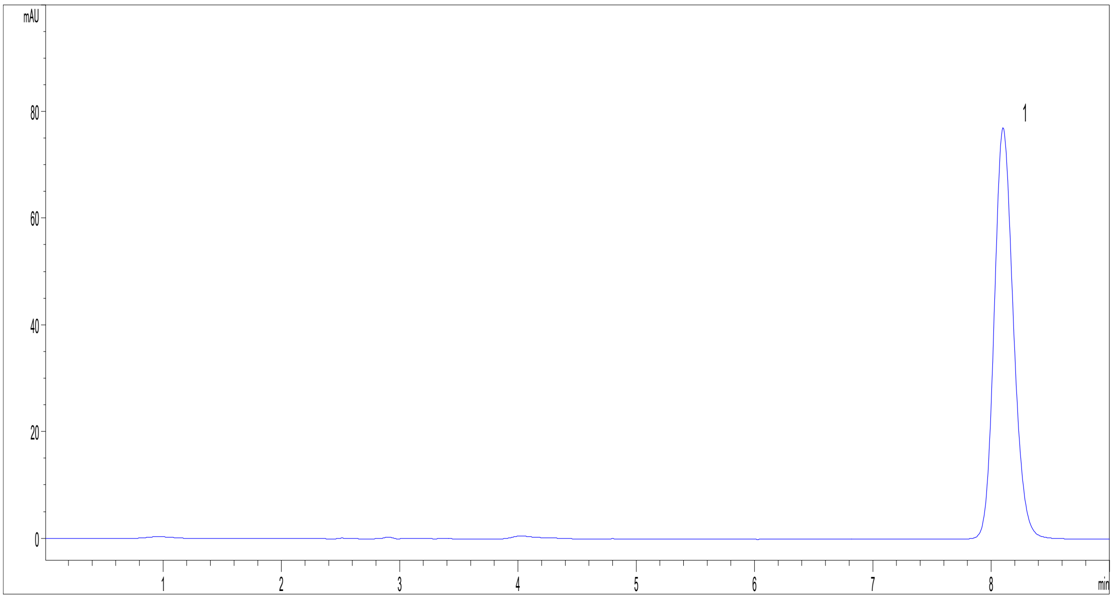

**FIGRURE 5 | ** HPLC chromatograms of A: XFG methanol extract and B: Tussilagone reference

Shionone: Solvent system, 96% acetonitrile and 4% water, flow rate 1.0 ml/min, UV detection wavelength 200 nm

A

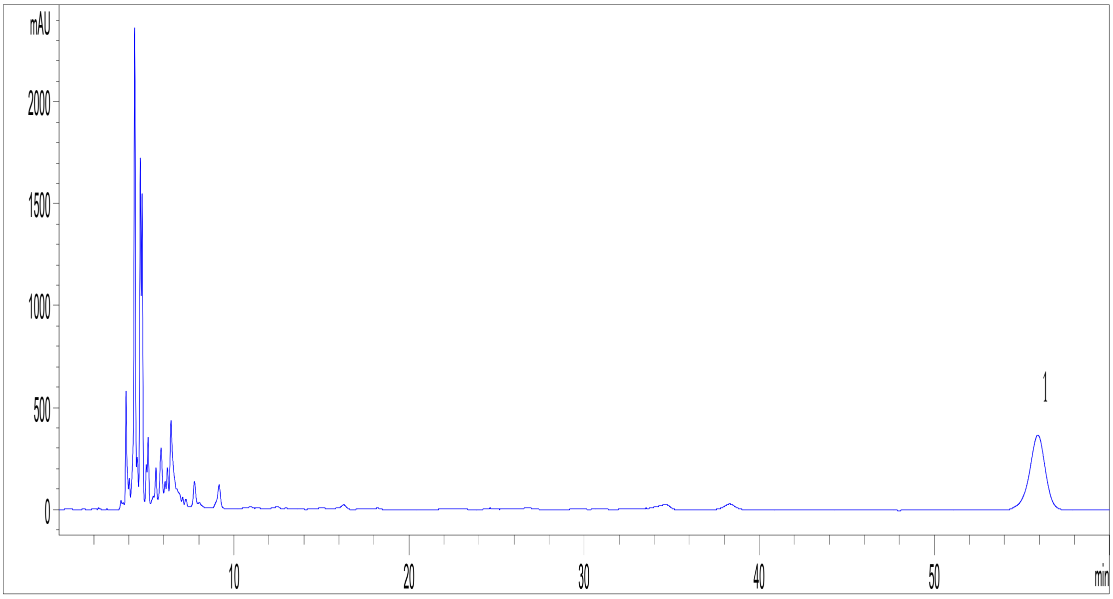

B

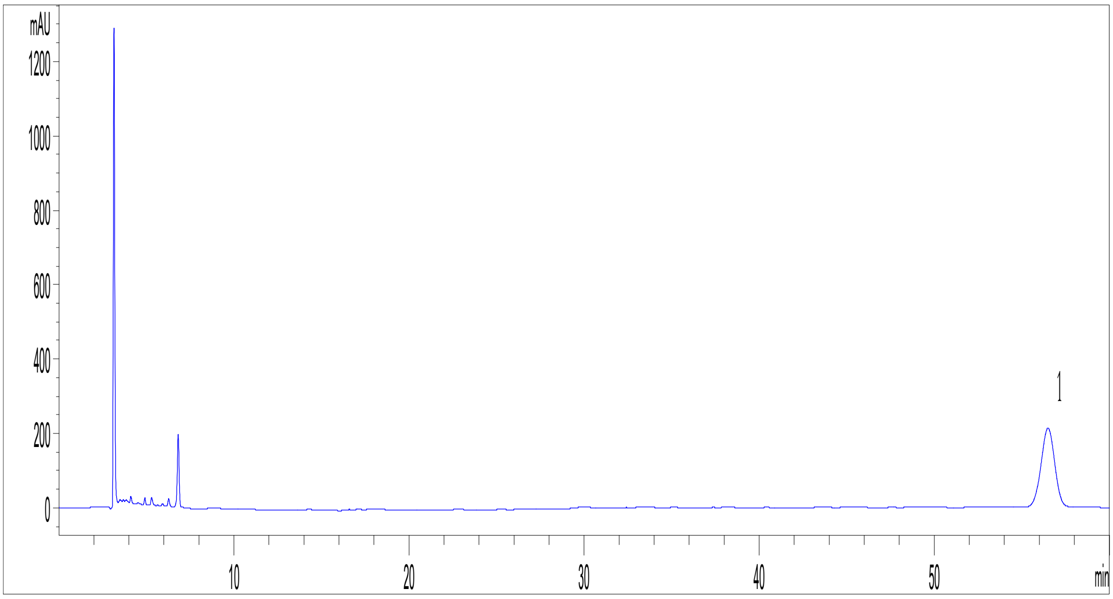

FIGRURE 6 | HPLC chromatograms of A: XFG methanol extract and B: Shionone reference

Atractylenolide III and Atractylenolide I: Solvent system, 80% methyl alcohol and 20% water, flow rate 1.0 ml/min, UV detection wavelength 220 nm

A

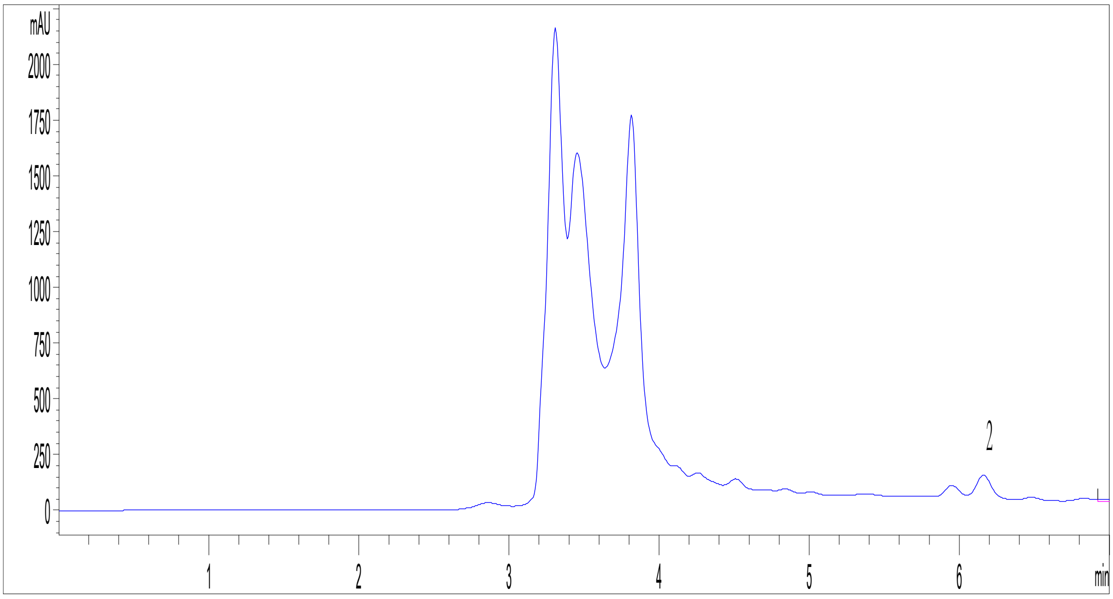

B

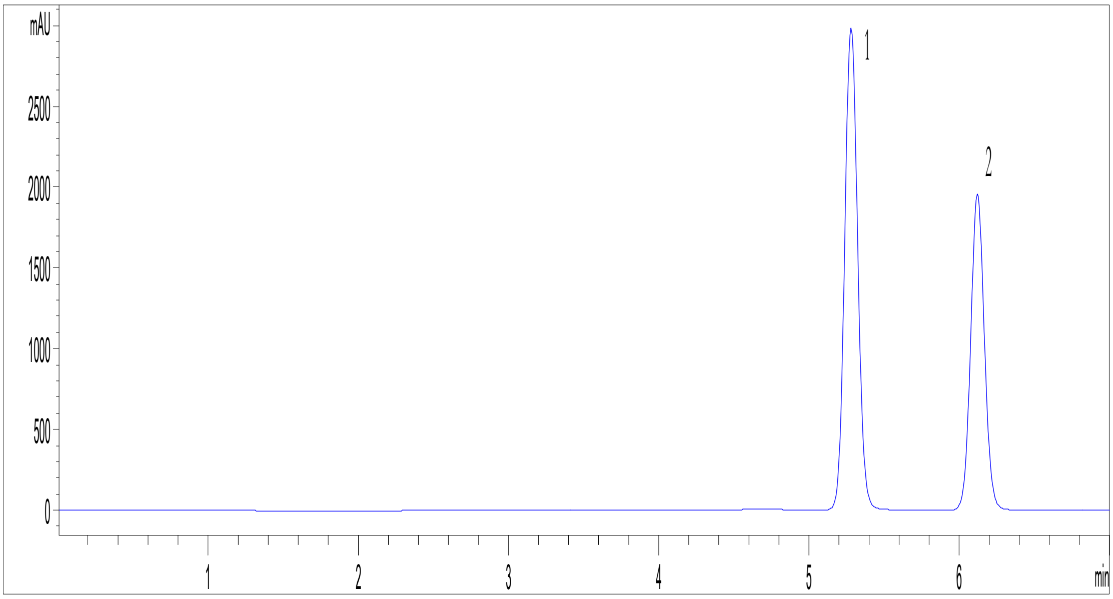

**FIGRURE 7 | ** HPLC chromatograms of A: XFG methanol extract, B1: Atractylenolide III and B2: Atractylenolide I reference

Chlorogenic acid: Solvent system, 75% acetonitrile and 25% water with 0.1% phosphoric acid, flow rate 1.0 ml/min, UV detection wavelength 330 nm

A

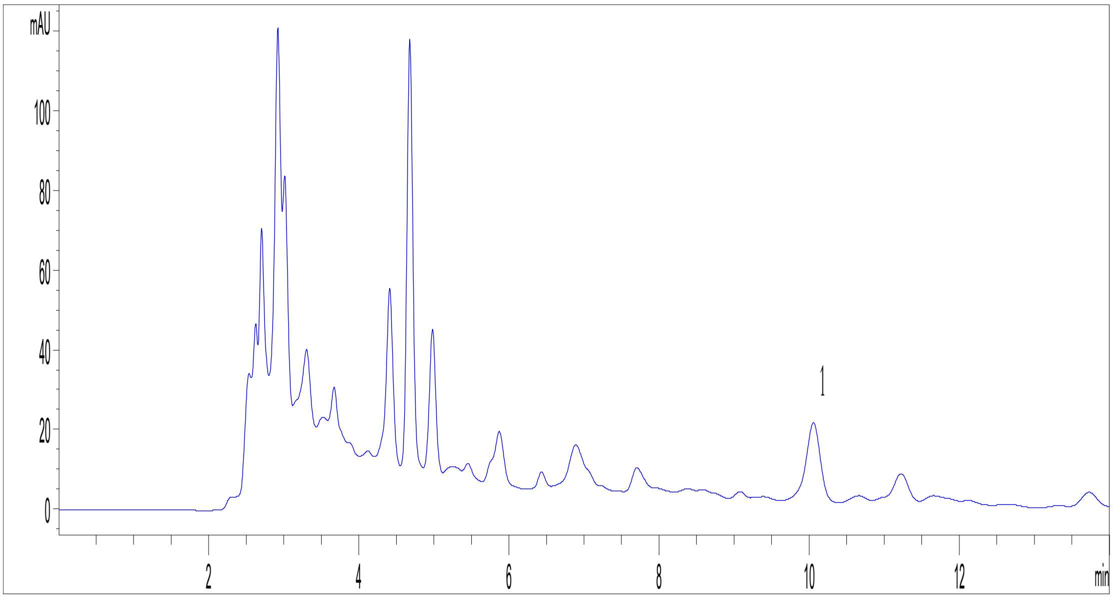

B

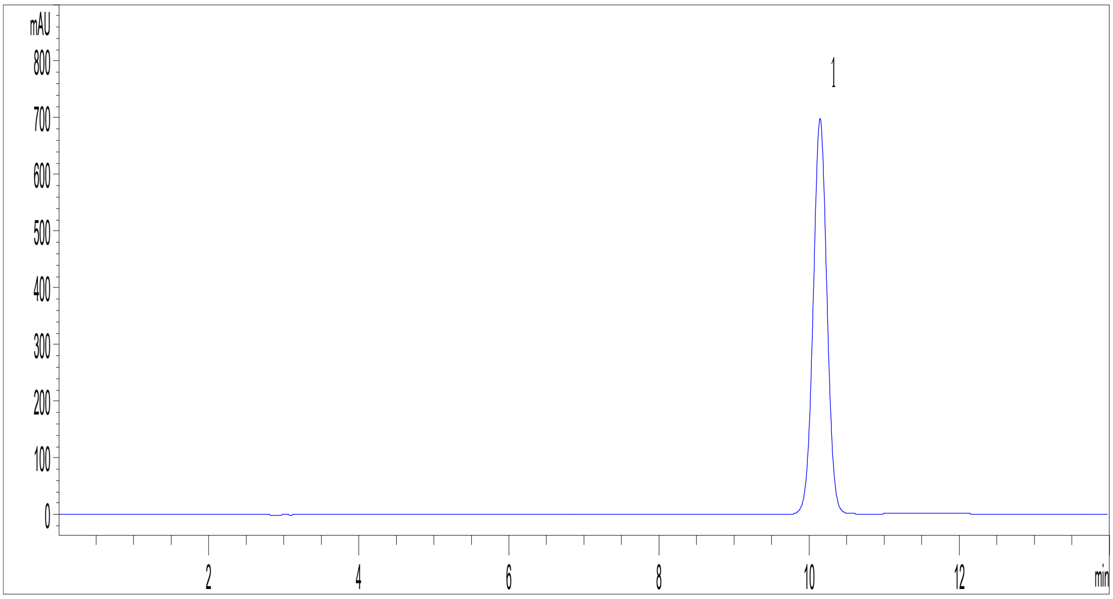

**FIGRURE 8 | ** HPLC chromatograms of A: XFG methanol extract and B: Chlorogenic acid reference

Scopoletin: Solvent system, 72% acetonitrile and 28% water with 0.1% phosphoric acid flow rate 1.0 ml/min, UV detection wavelength 355 nm

A

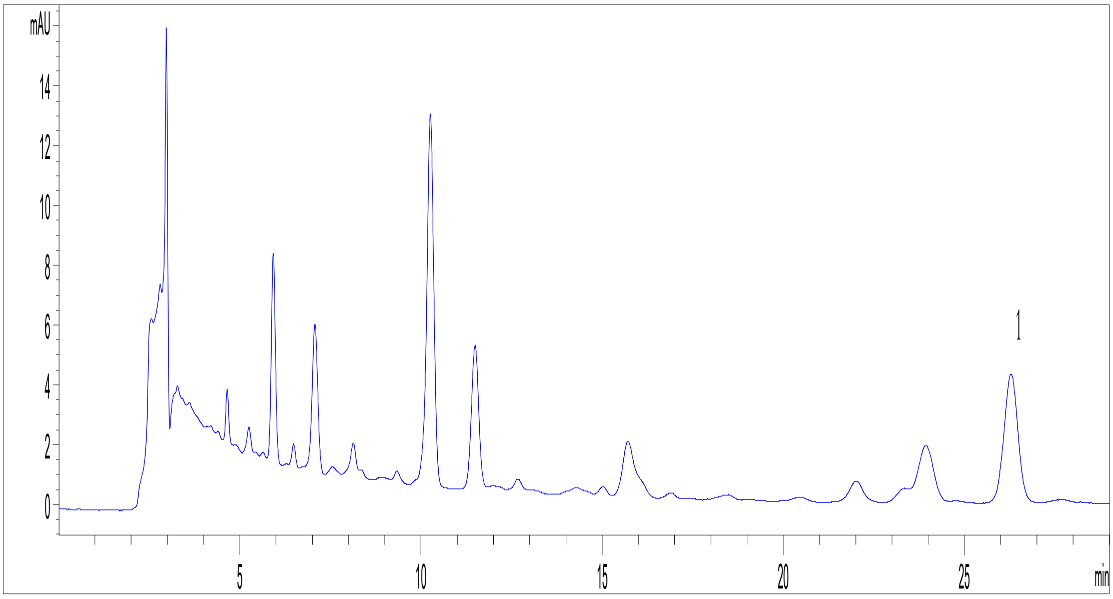

B

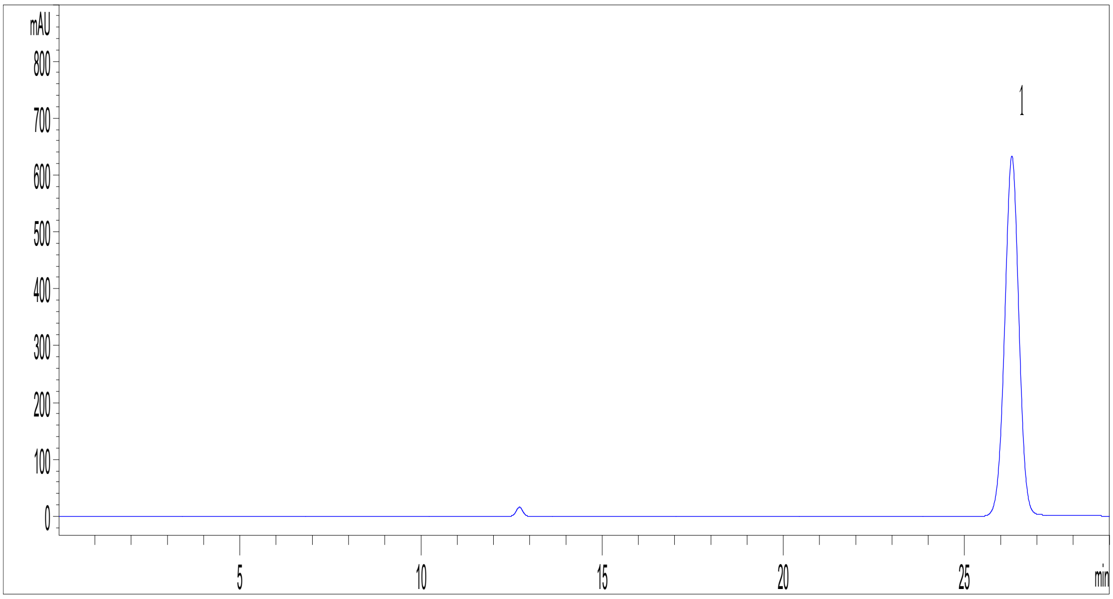

**FIGRURE 9 | ** HPLC chromatograms of A: XFG methanol extract and B: Scopoletin reference
