# Supplementary material for: Meta-Analysis on the Chinese Herbal Formula Xiaoer-Feike Granules as a Complementary Therapy for Children With Acute Lower Respiratory Infections
Source: Front Pharmacol. 2020 Oct 22;11:496348. doi: 10.3389/fphar.2020.496348 (PMC7642815; doi:10.3389/fphar.2020.496348)
Supplement: Supplementary file 2 [file DataSheet_2.pdf]

PHARMACOPOEIA
OF THE PEOPLE'S REPUBLIC OF CHINA

2015

Volume I

Chinese Pharmacopoeia Commission

China Medical Science Press

Xiao'er Feike Keli 1844
Xiao'er Feire Kechuan Koufuye 1846
Xiao'er Feire Kechuan Mixture 1846
Xiao'er Feireping Capsules 1847
Xiao'er Feireping Jiaonang 1847
Xiao'er Fuqi Zhixie Powder 1848
Xiao'er Fuqi Zhixie San 1848
Xiao'er Fuxiening Syrup 1848
Xiao'er Fuxiening Tangjiang 1848
Xiao'er Ganmao Cha 1850
Xiao'er Ganmao Granules 1852
Xiao'er Ganmao Keli 1852
Xiao'er Ganmao Koufuye 1852
Xiao'er Ganmao Medicinal Teas 1850
Xiao'er Ganmao Mixture 1852
Xiao'er Ganmaoning Syrup 1853
Xiao'er Ganmaoning Tangjiang 1853
Xiao'er Ganyan Granules 1849
Xiao'er Ganyan Keli 1849
Xiao'er Huadu Powder 1854
Xiao'er Huadu San 1854
Xiao'er Huashi Koufuye 1855
Xiao'er Huashi Mixture 1855
Xiao'er Huashi Pills 1856
Xiao'er Huashi Wan 1856
Xiao'er Jiebiao Granules 1857
Xiao'er Jiebiao Keli 1857
Xiao'er Jiegan Pian 1858
Xiao'er Jiegan Tablets 1858
Xiao'er Jiere Pills 1860
Xiao'er Jiere Wan 1860
Xiao'er Jindan Pian 1861
Xiao'er Jindan Tablets 1861
Xiao'er Jingfeng Powder 1861
Xiao'er Jingfeng San 1861
Xiao'er Kangxian Capsules 1862
Xiao'er Kangxian Jiaonang 1862
Xiao'er Kechuan Granules 1863
Xiao'er Kechuan Keli 1863
Xiao'er Qingfei Huatan Koufuye 1866
Xiao'er Qingfei Huatan Mixture 1866
Xiao'er Qingfei Zhike Pian 1867
Xiao'er Qingfei Zhike Tablets 1867
Xiao'er Qingre Pian 1869
Xiao'er Qingre Tablets 1869
Xiao'er Qingre Zhike Heji (Xiao'er Qingre Zhike Koufuye) 1870
Xiao'er Qingre Zhike Mixture 1870
Xiao'er Qixingcha Granules 1864
Xiao'er Qixingcha Keli 1864
Xiao'er Qixingcha Koufuye 1865
Xiao'er Qixingcha Mixture 1865
Xiao'er Resuqing Granules 1871
Xiao'er Resuqing Keli 1871
Xiao'er Resuqing Koufuye 1872
Xiao'er Resuqing Mixture 1872
Xiao'er Resuqing Syrup 1874
Xiao'er Resuqing Tangjiang 1874
Xiao'er Tuire Granules 1876
Xiao'er Tuire Heji (Xiao'er Tuire Koufuye) 1875
Xiao'er Tuire Keli 1876
Xiao'er Tuire Mixture 1875
Xiao'er Xaoshi Tablets 1880
Xiao'er Xiangju Pills 1877
Xiao'er Xiangju Wan 1877
Xiao'er Xiaoji Zhike Koufuye 1879
Xiao'er Xiaoji Zhike Mixture 1879
Xiao'er Xiaoshi Pian 1880
Xiao'er Xieli Pian 1881
Xiao'er Xieli Tablets 1881
Xiao'er Xiesuting Granules 1882
Xiao'er Xiesuting Keli 1882
Xiao'er Yanbian Granules 1883
Xiao'er Yanbian Keli 1883
Xiao'er Zhibao Pills 1886
Xiao'er Zhibao Wan 1886
Xiao'er Zhike Syrup 1884
Xiao'er Zhike Tangjiang 1884
Xiao'er Zhisou Syrup 1885
Xiao'er Zhisou Tangjiang 1885
Xiaochaihu Capsules 1833
Xiaochaihu Effervescent Tablets 1835
Xiaochaihu Granules 1834
Xiaochaihu Jiaonang 1833
Xiaochaihu Keli 1834
Xiaochaihu Paotengpian 1835
Xiaochaihu Pian 1836
Xiaochaihu Tablets 1836
Xiaocuo Pills 1804
Xiaocuo Wan 1804
Xiaohuoluo Pills 1888
Xiaohuoluo Wan 1888
Xiaojianzhong Granules 1889
Xiaojianzhong Heji 1888
Xiaojianzhong Keli 1889
Xiaojianzhong Mixture 1888
Xiaojianzhong Pian 1890
Xiaojianzhong Tablets 1890
Xiaojin Capsules 1892
Xiaojin Jiaonang 1892
Xiaojin Pian 1893
Xiaojin Pills 1894
Xiaojin Tablets 1893
Xiaojin Wan 1894
Xiaoke Pills 1808
Xiaoke Wan 1808
Xiaokechuan Tangjiang 1805
Xiaokeling Pian 1806
Xiaokeling Tablets 1806
Xiaokeping Pian 1806
Xiaokeping Tablets 1806
Xiaoluotong Capsules 1809
Xiaoluotong Jiaonang 1809
Xiaoluotong Pian 1810
Xiaoluotong Tablets 1810
Xiaomi Shuan 1810
Xiaomi Suppositories 1810
Xiaoqinglong Granules 1896
Xiaoqinglong Heji 1895
Xiaoqinglong Keli 1896
Xiaoqinglong Mixture 1895
Xiaoshi Tuire Syrup 1811
Xiaoshi Tuire Tangjiang 1811
Xiaoshuan Granules 1812
Xiaoshuan Keli 1812
Xiaoshuan Koufuye 1813
Xiaoshuan Mixture 1813
Xiaoshuan Tongluo Capsules 1814
Xiaoshuan Tongluo Granules 1815
Xiaoshuan Tongluo Jiaonang 1814
Xiaoshuan Tongluo Keli 1815
Xiaoshuan Tongluo Pian 1817

reference to the peak of forsythin.

Reference solution Dissolve a quantity of forsythin CRS, accurately weighed, in methanol to produce a solution containing 0.1 mg of forsythin per ml.

Test solution Pulverize the contents obtained under the test of packing variation, weigh accurately about 2 g in a stoppered conical flask, add accurately 25 ml of methanol, stopper and weigh, ultrasonicate (power 300 W, frequency 50 kHz) for 30 minutes, allow to cool and weigh again, replenish the loss of weight with methanol, mix well and filter, Measure accurately 10 ml of the successive filtrate, evaporate to dryness, dissolve the residue with 5 ml of 70% ethanol, apply to a neutral aluminium oxide column (100-200 mesh, 2 g, 1.5 cm in diameter), elute with 80 ml of 70% ethanol, collect the eluates, evaporate to dryness, dissolve the residue with 50% methanol, transfer to a 5 ml volumetric flask, dilute to volume with 50% methanol, mix well and filter, use the successive filtrate as the test solution.

Procedure Inject accurately 10 μl of each of the reference solution and test solution into the column, determine and calculate the content.

It contains not less than 0.68 mg of forsythin ($C_{27}H_{34}O_{11}$) per pack for [Strength (1) and (3)], 1.36 mg of forsythin ($C_{27}H_{34}O_{11}$) per pack for [Strength (2) and (4)], referred to Forsythiae Fructus.

Actions To disperse wind, release the exterior, clear heat and remove food stagnation.

Indications Pattern of wind-heat common cold with food stagnation, manifested as fever, cough, runny nose, runny nose, red and swelling throat, poor appetitte, distention and fullness in the epigastrium and abdomen, constipation or sour fetid stool, and deep yellow urine.

Administration and Dosage Take the medicine orally after mixing it with hot water, 1-2 g per time for children between 6 months and 1 year old; 2-3 g per time for 1 to 3 year-old children, 3-4 g per time for 4 to 6 year-old children; 4-5 g per time for 7 to 9 year-old children; 6 g per time for children more than 10 years old. Three times a day.

Strength (1) 2 g per pack
(2) 4 g per pack
(3) 2 g per pack (without sucrose)
(4) 4 g per pack (without sucrose)

Storage Preserve in tightly closed containers.

Xiao'er Feike Keli
(小儿肺咳颗粒)
Xiao'er Feike Granules

Ingredients Ginseng Radix et Rhizoma 20 g; Poria 20 g; Macrocephalae Rhizoma 8 g; Citri Pericarpium Reticulatae 20 g; Galli Gigerii Endothelium Corneum 20 g; Rhei Radix et Rhizoma (processed with wine) 12 g; Trionycis Carapax 20 g; Lycii Cortex 23 g; Glehniae Radix 39 g; Glycyrrhizae Radix et Rhizoma 12 g; Artemisiae Annuae Herba 29 g; Ophiopogonis Rradix 39 g; Cinnamomi Ramulus 8 g; Zingiberis Rhlzoma 8 g; Aconiti Lateralis Radix Praeparata 8 g; Trichosanthis Fructus 29 g; Farfarae Flos 20 g; Asteris Radix et Rhizoma 20 g; Cortex Mori 23 g; Arisaema Cum Bile 8 g; Astragali Radix 20 g; LyciiFructus 20 g.

Procedure Decoct Astragali Radix, Lycii Cortex, Glehniae Radix, Ophiopogonis Radix, Glycyrrhizae Radix et Rhizoma, Artemisiae Annuae Herba, Cinnamomi Ramulus, Trichosan-thisFructus, Asteris Radix et Rhizoma and Cortex Mori with water twice, 2 hours for each time, combine the decoctions, filter and concentrate to form a thin extract with a relative density of 1.26-1.30 (80℃). Pulverize the other ingredients to fine powder, mix thoroughly with the above thin extract and a quantity of sucrose, make granules dry and make to 1000 g.

Description Yellowish-brown to dark brown granules; taste, sweet.

Identification (1) Microscopical: Irregular branched masses colourless, dissolved gradually on mounting in chloral hydrate solution; hyphae colourless or pale brown (Poria). Pollen granules spheroidal, 28-40 μm in diameter, showing spiny sculptures on the exine, spines relatively sharped (Farfarae Flos). Stone cells of testa irregular polygonal in surface view, anticlinal walls deep sinuous or slightly waved, with distinct strations (Lycii Fructus).

(2) Pulverize 50 g of the granules, add 120 ml of methanol, heat under reflux for 2 hours, filter, and evaporate the filtrate to dryness, dissolve the residue in 30 ml of 7% solution of sulfuric acid, and heat under reflux for 1 hour, extract with three 20-ml quantities of petroleum ether (60-90℃) by shaking, and combine the petroleum ether extract. Wash with three 20-ml quantities of water, combine the petroleum ether extract, evaporate to dryness, and dissolve the residue in 1 ml of dehydratedethanol as the test solution. Prepare a solution with 1 g of Ginseng Radix et Rhizoma reference drug in 30 ml of methanol in the same manner as the reference drug solution. Dissolve panaxotriol CRS in ethanol to produce a solution containing 1 mg per ml as the reference solution. Carry out the method for thin layer chromatography (*0502*), using silica gel G as the coating substance and a mixture of toluene and acetone (2 : 1) as the mobile phase. Apply separately 10 μl of each of the above three solutions to the plate. After developing, remove the plate, dry in the air. Spray with a solution of sulfuric acid in ethanol (1→10), heat at 100℃ to the spots clear, and examine separately in daylight and under ultraviolet light at 365 nm. The spots in the chromatogram obtained with the test solution correspond in position and colour to the spots in the chromatogram obtained with the reference drug solution and reference solution in day light. Examine under ultraviolet light, the same fluorescent spots are shown.

(3) Pulverize 15 g of the granules, ultrasonicate in 50 ml of *n*-butanol saturated with water for 30 minutes, filter, and wash the filtrate with three 20-ml quantities of water, and discard the washings. Evaporate the *n*-butanol extract to dryness, and dissolve the residue in 1 ml of methanol as the test solution. Weigh 0.5 g of Atractylodis Macrocephalae Rhizoma reference drug, decoct with water for 2 hours, filter, extract the filtrate with three 15-ml quantities of *n*-butanol saturated with water by shaking, combine the *n*-butanol extract, evaporate the *n*-butanol extract to dryness, and dissolve the residue in 1 ml of methanol as the reference drug solution. Carry out the method for thin layer chromatography (*0502*), using silica gel G as the coating substance and a mixture of chloroform, acetone and formic acid (9.5 : 0.5 : 0.06) as the mobile phase. Apply separately 5 μl of each of the above two solutions to the plate. After developing, remove the plate, dry in the air. Examine under ultraviolet light at 365 nm. A blue fluorescent spot in the chromatogram obtained with the test solution corresponds in position and colour to the blue fluorescent spot in the chromatogram obtained with the

reference drug solution.

(4) Pulverize 10 g of the granules, add 30 ml of methanol, heat under reflux for 1 hour, filter, and evaporate the filtrate to dryness, dissolve the residue in 4 ml of methanol, filter and use the filtrate as the test solution. Dissolve synephrine CRS in methanol to produce a solution containing 1 mg per ml as the reference solution. Carry out the method for thin layer chromatography 〈*0502*〉, using silica gel G as the coating substance and a mixture of chloroform, acetone, methanol and concentrated ammonium TS (13 : 4 : 3 : 0.5) as the mobile phase. Apply separately 5 μl of each of the above two solutions to the plate. After developing, remove the plate, dry in the air. Spray with a 0.5% solution of ninhydrine in ethanol, heat at 105℃ to the spots clear and examine in daylight. The spot in the chromatogram obtained with the test solution corresponds in position and colour to the spot in the chromatogram obtained with the reference solution.

(5) Pulverize 10 g of the granules, ultrasonicate in 30 ml of chloroform for 20 minutes, filter, and evaporate the filtrate to dryness, dissolve the residue in 1 ml of chloroform as the test solution. Weigh 0.5 g of Artemisiae Annuae Herba reference drug, decoct with water for 30 minutes, filter, and evaporate the filtrate to dryness, dissolve the residue in 20 ml of chloroform by stirring, filter, and evaporate the filtrate to dryness, and dissolve the residue in 1 ml of chloroform as the reference drug solution. Carry out the method for thin layer chromatography 〈*0502*〉, using silica gel G as the coating substance and a mixture of chloroform and methanol (18 : 0.5) as the mobile phase. Apply separately 5 μl of each of the above two solutions to the plate. After developing, remove the plate and dry in air. Examine under ultraviolet light at 365 nm. A blue fluorescent spot in the chromatogram obtained with the test solution corresponds in position and colour to the blue fluorescent spot in the chromatogram obtained with the reference drug solution.

(6) Pulverize 5 g of the granules, ultrasonicate in 20 ml of methanol for 20 minutes, filter, and evaporate the filtrate to dryness. Dissolve the residue in 10 ml of water, add 1 ml of hydrochloric acid, heat on water bath for 30 minutes, cool immediately, extract with two 20-ml quantities of ether by shaking, and combine the ether extract. Evaporate the ether extract to dryness, and dissolve the residue in 1 ml of chloroform as the test solution. Prepare a solution with 0.1 g of Rhei Radix et Rhizoma reference drug in the same manner as the reference drug solution. Dissolve emodin CRS in methanol to produce a solution containing 1 mg per ml as the reference solution. Carry out the method for thin layer chromatography 〈*0502*〉, using silica gel G as the coating substance and the upper layer of a mixture of petroleum ether (30-60℃), ethyl formate and formic acid (15 : 5 : 1) as the mobile phase. Apply separately 3 μl of each of the above three solutions to the plate. After developing, remove the plate, dry in the air. Examine under ultraviolet light at 365 nm. Five orange fluorescent spots in the chromatogram obtained with the test solution correspond in position and colour to the fluorescent spots in the chromatogram obtained with the reference drug solution. The orange fluorescent spot in the chromatogram obtained with the test solution corresponds in position and colour to the orange fluorescent spot in the chromatogram obtained with the reference solution. Upon exposure to ammonia vapour, the spot turns red in daylight.

(7) Pulverize 50 g of the granules, ultrasonicate in 100 ml of methanol for 1 hour, filter, and evaporate the filtrate to dryness, dissolve the residue in 50 ml of water, extract with four 50-ml quantities of ethyl acetate by shaking, and combine the ethyl acetate extract. Evaporate the ethyl acetate extract to dryness, and dissolve the residue in 1 ml of methanol as the test solution. Weigh 1 g of Farfarae Flos reference drug, add 20 ml of methanol, ultrasonicate for 30 minutes, filter, and concentrate the filtrate to 1 ml as the reference drug solution. Carry out the method for thin layer chromatography 〈*0502*〉, using silica gel GF_{254} as the coating substance and a mixture of petroleum ether (60-90℃) and acetone (6 : 1) as the mobile phase. Apply separately 5 μl of each of the above two solutions to the plate. After developing, remove the plate, dry in the air. Examine under ultraviolet light at 254 nm. The fluorescent spots in the chromatogram obtained with the test solution correspond in position and colour to the fluorescent spots in the chromatogram obtained with the reference drug solution.

(8) Weigh 1 g of Lycii Fructus reference drug, add 30 ml of methanol, ultrasonicate for 30 minutes, filter, and concentrate the filtrate to 1 ml as the reference drug solution. Carry out the method for thin layer chromatography 〈*0502*〉, using silica gel G as the coating substance and a mixture of ethyl acetate, chloroform and formic acid (3 : 3 : 1) as the mobile phase. Apply separately 5 μl of the test solution obtained under *Identification* (*7*) and the reference drug solution to the plate. After developing, remove the plate, dry in the air. Examine under ultraviolet light at 365 nm. The fluorescent spots in the chromatogram obtained with the test solution correspond in position and colour to the fluorescent spots in the chromatogram obtained with the reference drug solution.

Limit test for aconitine Pulverize a quantity of the containing to fine powder, weigh 36 g of the powder to a stoppered conical flask, moisten with 10 ml of concentrated ammonia TS, add 80 ml of chloroform, ultrasonicate for 30 minutes, filter and the filtrate evaporated to dryness. Dissolve the residue in 30 ml 3% solution of sulfuric acid, extract the filtrate with three 40 ml quantities of chloroform by shaking, discard the chloroform solution, adjust pH of the water layer to 10 with concentrated ammonia TS, and extract with five 30-ml quantities of chloroform. Combine and recover solvent, dissolve the residue with a quantity of chloroform, transfer to a 2 ml volumetric flask, dilute to the volume and mix well as the test solution. Dissolve aconitine CRS in chloroform to produce a solution containing 1.5 mg per ml as the reference solution. Carry out the method for thin layer chromatography 〈*0502*〉, using silica gel G as the coating substance and a mixture of cyclohexane, ethyl acetate and methanol (6.4 : 3.6 : 1) as the mobile phase. Apply separately 5 μl of the test solution and the reference solution to the plate. After developing in the chamber pre-equilibriated for 2 hours and removal of the plate, dry in air, spray with diluted solution of potassium hepta-iodobismuthate TS. The spots in the chromatogram obtained with the test solution are smaller or no in position and colour than the spots in charomatogram obtained with the reference solution.

Other requirements Comply with the general requirements for granules 〈*0104*〉.

Assay Carry out the method for high performance liquid chromatography 〈*0512*〉.

Chromatographic system and system suitability Use octadecylsilane bonded silica gel as the stationary phase and a mixture of methanol and 5% acetic acidsolution (33 : 67) as the mobile phase. As detector a spectrophotometer set at 283 nm. The number of theoretical plates of the column is
